# Gut microbiota composition in depressive disorder: a systematic review, meta-analysis, and meta-regression

**DOI:** 10.1038/s41398-023-02670-5

**Published:** 2023-12-08

**Authors:** Mingxue Gao, Jizhi Wang, Penghong Liu, Hongwei Tu, Ruiyu Zhang, Yanyan Zhang, Ning Sun, Kerang Zhang

**Affiliations:** 1 Department of Psychiatry, First Hospital of Shanxi Medical University, 030001 Taiyuan, China; 2 First Clinical Medical College, Shanxi Medical University, 030001 Taiyuan, China; 3 Basic Medical College, Shanxi Medical University, 030001 Taiyuan, China

**Keywords:** Depression, Predictive markers

## Abstract

Studies investigating gut microbiota composition in depressive disorder have yielded mixed results. The aim of our study was to compare gut microbiome between people with depressive disorder and healthy controls. We did a meta-analysis and meta-regression of studies by searching PubMed, Web of Science, Embase, Scopus, Ovid, Cochrane Library, ProQuest, and PsycINFO for articles published from database inception to March 07, 2022. Search strategies were then re-run on 12 March 2023 for an update. We undertook meta-analyses whenever values of alpha diversity and Firmicutes, Bacteroidetes (relative abundance) were available in two or more studies. A random-effects model with restricted maximum-likelihood estimator was used to synthesize the effect size (assessed by standardized mean difference [SMD]) across studies. We identified 44 studies representing 2091 patients and 2792 controls. Our study found that there were no significant differences in patients with depressive disorder on alpha diversity indices, Firmicutes and Bacteroidetes compared with healthy controls. In subgroup analyses with regional variations(east/west) as a predictor, patients who were in the West had a lower Chao1 level (SMD −0.42[−0.74 to −0.10]). Subgroup meta-analysis showed Firmicutes level was decreased in patients with depressive disorder who were medication-free (SMD −1.54[−2.36 to −0.72]), but Bacteroidetes level was increased (SMD −0.90[0.07 to 1.72]). In the meta-regression analysis, six variables cannot explain the 100% heterogeneity of the studies assessing by Chao1, Shannon index, Firmicutes, and Bacteroidetes. Depleted levels of *Butyricicoccus, Coprococcus, Faecalibacterium, Fusicatenibacter, Romboutsia*, and enriched levels of *Eggerthella, Enterococcus, Flavonifractor, Holdemania, Streptococcus* were consistently shared in depressive disorder. This systematic review and meta-analysis found that psychotropic medication and dietary habit may influence microbiota. There is reliable evidence for differences in the phylogenetic relationship in depressive disorder compared with controls, however, method of measurement and method of patient classification (symptom vs diagnosis based) may affect findings. Depressive disorder is characterized by an increase of pro-inflammatory bacteria, while anti-inflammatory butyrate-producing genera are depleted.

## Introduction

Depressive disorder, a debilitating psychiatric disorder, is the leading cause of disability worldwide [1]. The World Health Organization (2019) estimates the global loss in productivity due to depressive disorders amounts to $1 trillion per year –a trajectory expected to rise [2]. Compositional variations or other dysregulations of the gut microbiome are increasingly believed to play key roles in the pathogenesis of mental illnesses [3–8]. The gut microbiome can potentially affect the brain through multiple pathways such as inducing metabolites of tryptophan [9, 10], stimulation of the vagal nerve [11, 12], inducing alterations of the hypothalamus-pituitary-adrenal axis [13], microbial production of human neurotransmitters [12], and stimulation of the immune system over a gut epithelium with higher permeability [14, 15] altogether summarized in the concept of the gut-brain axis [16], a bidirectional communication system between the gastrointestinal tract (GI) and the central nervous system (CNS) [17].

Several animal studies [11, 18–22] have consistently revealed that when the microbiome is transplanted from patients with depressive disorder to normal animals, depressive-like behaviors are induced, whereas transplantation from healthy hosts improves depressive symptoms. This demonstrates depression as a causal factor for microbiota change. Clinical studies [23–54] investigating the association between gut microbiome and depressive disorder have yielded mixed results. These inconsistent findings might be attributable to the use of psychotropic medication, country of study, age, sex and body mass index (BMI) of depressed patients, severity of depressive symptoms. Only two systematic reviews [55, 56] incorporated a meta-analysis of alpha diversity. Owing to the small number of studies included, Sanada et al. (2020) did not analyze the effects of confounders [55]. Nikolova et al. (2020) aimed at finding distinct or shared gut microbial alterations in psychiatric disorders, although they analyzed alpha and beta diversity and relative abundance of gut microbes in major depressive disorder (MDD), they did not analyze the effects of confounders in MDD [56].

The aim of our study was to compare gut microbiome between people with depressive disorder and healthy controls, using a systematic review and meta-analysis of available studies in the scientific literature. We will also explore sources of heterogeneity between studies using subgroup meta-analysis and meta-regression.

## Methods

### Search strategy and selection criteria

The protocol for this review was preregistered with PROSPERO (CRD42022315694). This systematic review and meta-analysis was conducted in adherence with the Preferred Reporting Items for Systematic Reviews and Meta-Analyses (PRISMA) guidelines [57]. Eight electronic databases including PubMed, Web of Science, Embase, Scopus, Ovid, Cochrane Library, ProQuest, and PsycINFO were searched on 7 March 2022 using the key terms “(depressive disorder OR depressive syndrome OR unipolar depression) AND (gastrointestinal microbiome OR gut microbiome OR gut microflora OR gut microbiota OR gastrointestinal flora OR gut flora OR gastrointestinal microbiota OR gastric microbiome OR intestinal microbiome)”. To describe the concept of implementation, Medical Subject Heading (MeSH) terms were used in PubMed and Emtree terms were used in Embase. We did not apply any restrictions on study design or publication data. To identify other potentially relevant studies, the reference list of reviews that were excluded from this study was manually searched [55, 58–63]. Search strategies were then re-run on 12 March 2023 for an update.

Titles and abstracts were independently screened by two authors (MG and JW) to identify possible articles for full-text retrieval. Inconsistencies in screening decisions were solved by consulting a third author (KZ). Firstly, titles and abstracts resulting from the search strategy were selected if they met the following inclusion criteria: (i) cross-sectional studies or reported baseline data from longitudinal studies of gut microbiota composition comparing patients who had depressive disorder with healthy controls, (ii) performed gut microbiota analysis and reported diversity or abundance measures, and (iii) published as full-text articles in peer-reviewed scientific journals. Correspondingly, the exclusion criteria were: (i) they examined the gut microbiota and anxiety/depression/bipolar disorder symptoms solely in another psychiatric disorder or disease, (ii) assessed the effect of an intervention without reporting relevant baseline measurements, (iii) no healthy controls, and (iv) published as reviews, case reports, conference abstracts, or letters. Next, the full text of relevant papers was then assessed for eligibility for inclusion using the same criteria.

### Data extraction

Data were extracted and cross-checked by two independent authors (MG and JZW) using a predesigned template. We used Endnote to remove duplicate data. Information gathered for each study included the following items: name of the first author, publication year, the country in which the study was in, sample size, definition of disorder, age, sex (%female), body-mass index (BMI), smokers, alcohol, medication (such as antipsychotics, mood stabilizers, and antidepressants), Hamilton Depression Rating Scale (HDRS) scores, sequencing, diversity assessments, and methodological information. As primary outcomes of interest, we extracted community-level measures of gut microbiota composition (alpha and beta diversity) and taxa composition at phylum, order, family, and genus levels (relative abundance). Alpha diversity provides a summary statistic of the microbial community, whereby higher alpha diversity indicates a greater number of species (i.e., “richness”), with more even representation (i.e., “evenness”), and/or greater biodiversity according to the ancestral dissimilarity of species (i.e., “phylogenetic diversity”) [64, 65]. Beta diversity is an inter-individual measure that examines the similarity of communities relative to the other samples analyzed [66].

### Data analysis

To evaluate the quality of the studies, two authors (MG and JW) independently used the Newcastle-Ottawa Scale (NOS) for the observational studies [67, 68]. The tool was developed to assess the quality of nonrandomized studies with its design, content, and ease of use directed to the task of incorporating the quality assessments in the interpretation of meta-analytic results. A ‘star system’ has been developed in which a study is judged on three broad perspectives: the selection of the study groups; the comparability of the groups; and the ascertainment of either the exposure or outcome of interest for case-control or cohort studies respectively. In cases of disagreement between the authors in some aspects of the evaluation, a third reviewer (KZ) was consulted to make the final decision.

The systematic review and meta-analysis consisted of three steps. First, we did the overall analysis for two between-group meta-analyses comparing alpha diversity in patients with depression, with those in healthy controls (cross-sectional studies). Additionally, we did a prespecified subgroup analysis of patients in depression by psychotropic medication and regional variations. We did subgroup analyses for these two specific moderators in view of their well-known effects on gut microbiota and their clinical implications [69–74]. We did meta-regression analyses to investigate possible moderators of alpha diversity. Restricted maximum likelihood random-effects meta-regressions [75, 76] of effect size were done with regional variations(east/west), use of psychotropic medication, mean age, sex, BMI, and severity of disease (as assessed by HDRS) as moderators. Studies were weighted such that the studies with the most precise parameters, quantified by the sample size and 95% confidence interval (CI), had more influence in the regression analyses.

Because studies used different measurement methods, we used standardized mean difference estimates of the differences in alpha diversity between patients and healthy controls as the effect size. A random-effects meta-analysis on Cohen’s d standardized mean difference (SMD) was performed applying the inverse-variance method, which allows population-level inferences and is more stringent than fixed-effect models [77, 78], and also calculated the corresponding 95% CIs. Random-effect modeling assumes a genuine diversity in the results of various studies and incorporates a between-study variance into the calculations [78]. The effect size was categorized as a low effect (cut-off level 0.2), meaning a small difference in alpha diversity between patients and controls, a moderate effect (cut-off level 0.5), and a large effect (cut-off level 0.8) [79]. The direction of the effect size was positive if patients with depressive disorder had increased alpha diversity, and negative if they had decreased alpha diversity compared with controls in the between-group meta-analyses. We assessed heterogeneity across studies using the Cochran Q test, a weighted sum of the squares of the deviations of individual study effect size estimates from the overall estimate, and considered a p value of less than 0.10 significant [80]. Inter-study heterogeneity was quantified using the DerSimonian–Laird estimator, reported with the *I*^2^ statistic and interpreted the percentage of total variation across several studies as a result of heterogeneity, and heterogeneity was considered moderate when *I*² is between 50% and 75%, and high when *I*² is greater than 75% [81]. We did sensitivity analyses to ascertain whether the results of our analyses were strongly influenced by any single study or a cluster of studies sharing some characteristic. The overall significance was recomputed after each study or group of studies with a common feature were deleted from the analysis. Publication bias was evaluated with funnel plots and Egger’s regression test [82, 83]. The level of significance for effect size estimates was set at *p* < 0.05.

Second, for beta diversity, we performed a qualitative synthesis in order to examine the differences of communities in depression compared with healthy control.

Third, for the relative abundance of microbial taxa, we performed a qualitative synthesis owing to the large number and limited overlap of findings. We summarized findings for each taxon reported in at least two studies and labeled those increased, decreased, or no difference. Because Firmicutes and Bacteroidetes are two major focuses in the human studies related to the gut microbiota and depression [84, 85], we conducted a quantitative synthesis. Medians and inter-quartile ranges were transformed to means (M) and standard deviations (SD) using a web-based tool [86]. Where necessary, numerical data were extracted from graphs using GetData Graph Digitizer (v.2.20) [87]. We used StataMP version 16.0 for all meta-analyses [78].

## Results

### Search results

**Fig. 1:**
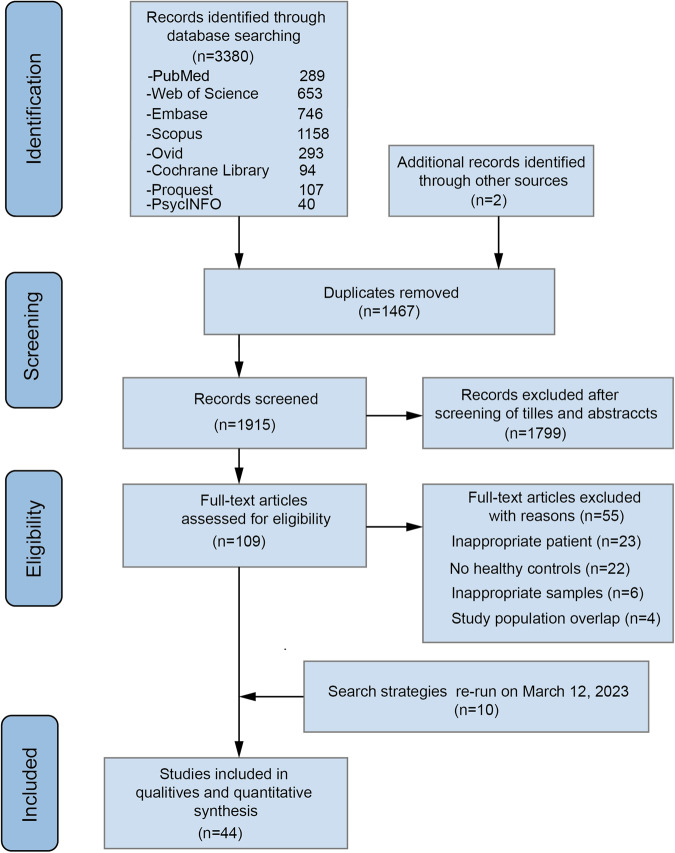
Study selection for meta-analysis of gut microbiota in depressive disorder.

The electronic search yielded 3380 papers. Additionally, 2 other records were identified as likely relevant to the review through other sources. The study selection process is presented in Fig. 1. Characteristics of included studies and quality of studies are described in the Tables 1 and S1 separately.

**Table 1:** Characteristics of studies included in the meta-analysis.

Study	Country	Definition of disorder	Scale score of patients	Sample size n	Mean age	% Female	Mean BMI	Smoking	Alcohol	Medicated	Sequencing	Diversity assessments
Aizawa et al. [23]	Japan	DSM-IV HDRS	16.9(6.8)	P: 43 HC: 57	P: 39.4 HC: 42.3	P: 41.9 HC: 61.4	P:23.2 HC: 22.2	Nr	Nr	Yes	16S rRNA-targeted RT-qPCR	α: not measured β: not measured
Bai et al. [24]	China	DSM-IV HDRS	25.3 (6.0)	P: 60 HC: 60	P: 35.6 HC: 35.1	P: 65.0 HC: 60.0	P: 20.9 HC: 21.2	Nr	No	No	16S rRNA Nr	α: Chao1., Shannon, Simpson, PD β: measured, not reported
Caso et al. [25]	Spain	DSM-IV-TR HDRS	21.17	P: 46 HC: 45	P: 42.1 HC: 44.7	P: 78.3 HC: 75.5	<30	Yes	Yes	Yes	16S rRNA V3-V4	α: Shannon β: Jaccard,Bray-Curtis
Chahwan et al. [26]	Australia	M.I.N.I BDI	28.4(9.9)	P: 71 HC: 20	P: 36.0 HC:36.0	P: 69.0 HC: 75.0	Nr	Yes	Yes	Yes	16S rRNA V3-V4	α: Observed sp., Chao1, Shannon β: UniFrac (weighted)
Chen et al. [28]	China	DSM-IV HDRS	25.6(4.7)	P: 10 HC: 10	P: 43.9 HC:39.6	P: 50.0 HC: 50.0	P: 23.5 HC: 22.6	No	No	Yes	Metaproteomics	α: not measured β: not measured
Chen et al. [27]	China	DSM-5 HDRS	28.0(4.6)	P: 62 HC: 46	P: 39.6 HC: 36.9	P: 100.0 HC: 100.0	P: 22.0 HC: 22.2	No	No	No	16S rRNA V3-V4 Shotgun Metagenomics	α: Observed sp., Chao1, ACE, Shannon, Simpson β: UniFrac (weighted & unweighted)
Chen et al. [88]	Taiwan	DSM-5 BDI	16.6 (14.6)	P: 10 HC: 10	P: 40.9 HC: 38.2	P: 80.0 HC: 70.0	P: 20.8 HC: 22.0	Nr	Nr	Yes	16S rRNA V4 and V3-V4	α: Richness, Shannon β: Bray-Curtis
Chung et al. [29]	Taiwan	DSM-5 BDI	19.2 (12.5)	P: 36 HC: 37	P: 45.8 HC: 41.2	P: 82.4 HC: 62.2	P: 22.8 HC: 24.0	Yes	Nr	Yes	16S rRNA V3-V4	α: Observed sp., Chao1, Shannon, PD β: UniFrac (weighted & unweighted)
Dong et al. [30]	China	DSM-5 HDRS	29.3(7.5)	P: 23 HC: 10	P:30.0 HC: 30.2	P: 69.6 HC: 60.0	P: 21.9 HC:21.5	Nr	No	No	16S rRNA V3-V4	α: ACE, Chao1, Simpson, Shannon β: Bray-Curtis
Dong et al. [89]	China	DSM-IV HDRS	28.3 (7.6)	P: 63 HC: 30	P: 28.3 HC: 29.2	P: 68.3 HC:66.7	P: 21.7 HC: 21.5	Yes	Yes	No	16S rRNA Nr	α: Chao1, Simpson, Shannon β: Bray-Curtis
Fontana et al. [31]	Italy	DSM-IV-TR	Nr	P: 34 HC: 20	P: 53.3 HC: 42.4	P: 70.6 HC: 35.0	P: 24.5 HC: 22.6	Yes	Yes	Yes	16S rRNA V3-V4	α: not measured β: not measured
Han et al. [90]	China	DSM-IV HDRS	Nr	P: 51 HC: 30	P: 27.1 HC: 29.2	P: 72.5 HC: 66.7	P: 21.6 HC: 21.5	Nr	Nr	No	16S rRNA V3-V4	α: Chao, ACE, Shannon, Simpson β: Bray–Curtis
Huang et al. [32]	China	ICD-10	Nr	P: 27 HC: 27	P: 48.7 HC: 42.3	P: 74.1 HC: 74.1	P: 23.8 HC: 23.4	Nr	Nr	No	16S rRNA V3-V4	α: Chao1, ACE, Shannon, PD β: UniFrac (weighted & unweighted)
Jiang et al. [33]	China	DSM-IV HDRS	29.8 (7.6)	P: 29 HC: 30	P: 25.3 HC: 26.8	P: 37.9 HC: 50.0	P: 20.3 HC: 19.6	Yes	Nr	Yes	16S rRNA V1-V3	α: Chao1, ACE, Shannon, Simpson, Evenness β: UniFrac (unweighted)
Kelly et al. [22]	Ireland	DSM-IV HDRS	19.5 (14.0)	P: 34 HC: 33	P: 45.8 HC: 45.8	P: 32.8 HC: 42.4	P: 26.2 HC: 24.6	Yes	Yes	Yes	16S rRNA Nr	α: Observed sp., Chao1, Shannon, PD β: UniFrac (weighted & unweighted), Bray-Curtis
Kim et al. [91]	Korea	CES-D	Nr	P:167 HC:1071	P: 44.0 HC:45.7	P: 55.1 HC:33.2	P: 23.1 HC:23.8	Nr	Nr	Nr	16S rRNA V3-V4	α: Observed ASVs., Pielou’s evenness, Shannon,PD β: Bray-Curtis, Jaccard, UniFrac (weighted & unweighted)
Kovtun et al. [92]	Russia	ICD-10 HDRS	21.3(3.6)	P: 36 HC:38	P: 30.9 HC:34.0	P: 47.2 HC:50.0	P: 22.4 HC:24.4	Nr	Nr	No	Metagenomics	α: Shannon β: not measured
Lai et al. [34]	China	DSM-5 HDRS	19.8 (3.0)	P: 26 HC: 29	P: 43.7 HC: 39.4	P: 69.2 HC: 55.2	P: 21.2 HC: 21.1	Nr	Nr	Yes	Shotgun Metagenomics	α: Shannon, Fisher β: Bray–Curtis
Li et al. [93]	China	ICD-10 HDRS	Nr	P: 40 HC: 22	P: 37.9 HC: 44.0	P: 62.5 HC:40.9	Nr	Yes	Yes	No	16S rRNA V3-V4	α: Chao, ACE, Shannon, Simpson β: not measured
Lin et al. [35]	China	DSM-IV-TR HDRS	Nr	P: 10 HC: 10	P: 36.2 HC: 38.1	P: 40.0 HC: 40.0	P: 23.8 HC: 24.2	Yes	Nr	Yes	16S rRNA V3-V4	α: not measured β: UniFrac (weighted)
Liu et al. [38]	China	DSM-IV SDS	Nr	P: 15 HC: 20	P: 44.8 HC: 43.9	P: 73.3 HC: 65.0	P: 22.0 HC: 24.6	Nr	Nr	No	16S rRNA V1-V3	α: Shannon β: not measured
Liu et al. [37]	USA	DSM-5 PROMIS	P:25.0 (6.9)	P: 43 HC: 47	P: 21.9 HC: 22.1	P: 88.4 HC: 72.3	Nr	No	Nr	Yes	16S rRNA V4	α: Observed sp., Shannon, PD β: UniFrac (weighted & unweighted), Bray-Curtis
Liu et al. [36]	China	DSM-IV HDRS	P:20.1(4.2)	P: 66 HC: 43	P: 24.2 HC: 23.7	P: 59.1 HC: 53.5	P: 21.5 HC: 21.8	Nr	Nr	No	16S rRNA V3-V4	α:Shannon, Simpson, Observed sp., Chao1, PD, Pielou’s evenness β:Jaccard
Mason et al. [39]	USA	DSM-IV QIDS-SR	15.6 (3.2)	P: 14 HC: 10	P: 41.9 HC: 33.0	P:78.6 HC:60.0	P: 31.0 HC: 25.6	Nr	No	Yes	16S rRNA V4	α: not measured β: UniFrac (weighted)
Naseribafrouei et al. [40]	Norway	ICD-10 MADRS	26.3 (7.6)	P: 37 HC: 18	P: 49.2 HC: 46.1	P:54.1 HC:61.1	P: 25.9 HC: 24.7	Nr	Nr	Nr	16S rRNA Nr	α: Observed sp., Simpsons β: not measured
Rong et al. [41]	China	DSM-5 HDRS	20.2 (3.1)	P: 31 HC: 30	P: 41.6 HC: 39.5	P: 71.0 HC:53.3	P: 21.5 HC: 22.0	Nr	Nr	Yes	Shotgun Metagenomics	α: Chao 1, Shannon, Inv. Simpson, Gm coefficient β: Bray-Curtis
Shen et al. [42]	China	M.I.N.I HDRS	Nr	P: 30 HC: 30	P: 44.8 HC: 44.0	P: 56.7 HC:50.0	P: 24.0 HC: 23.8	Yes	Yes	No	16S rRNA V3-V4	α:Ace, Chao1, Shannon, Simpson β:Jaccard
Stevens et al. [44]	USA	DSM-IV	Nr	P: 20 HC: 20	P: 34.0 HC: 34.0	P: 50.0 HC: 70.0	Nr	Nr	Nr	Yes	16S rRNA V3-V4	α: not measured β: Bray-Curtis
Stevens et al. [43]	USA	DSM-5	Nr	P: 7 HC: 21	P: 63.8 HC: 53.0	P: 57.1 HC: 61.9	P: 27.3 HC: 30.7	Nr	Nr	Nr	Shotgun Metagenomics	α: not measured β: not measured
Sun et al. [94]	China	DSM-IV HDRS	20.6(3.2)	P:31 HC: 29	P: 25.3 HC:24.8	P: 48.4 HC:55.2	P: 22.4 HC:22.0	Nr	No	No	16S rRNA V3-V4	α: Observed sp., Chao1, Simpson, Shannon, PD, Pielou’s evenness β: not measured
Thapa et al. [45]	USA	DSM-IV-TR BDI	Nr	P: 110 HC: 27	P: 19.5 HC: 20.3	P: 65.5 HC: 37.0	Nr	Nr	Yes	Yes	16S rRNA V4	α:Observed sp., Chao1, ACE, Shannon, PD β:UniFrac (weighted & unweighted), Bray-Curtis, Aitchison
Tsai et al. [95]	Taiwan	DSM-IV-TR HDRS	13.6(7.3)	P: 36 HC: 17	P: 65.6 HC: 64.1	P: 77.8 HC: 52.9	P: 23.4 HC: 24.5	Nr	Nr	Nr	16S rRNA V3-V4	α: Shannon, PD β:UniFrac (unweighted)
Valles-Colomer et al. [46]	Belgium	GP-reported depression	Nr	P: 80 HC: 70	P: 50.9	P: 54.6	P: 24.9	Nr	Nr	Yes	16S rRNA V4	α: not measured β: not measured
Yang et al. [47]	China	DSM-IV HDRS	22.2	P: 156 HC: 155	P: 29.6 HC: 29.1	P: 64.1 HC: 58.7	P: 22.3 HC: 22.4	Nr	Nr	Yes	Shotgun Metagenomics	α: Chao1, Shannon, Inv. Simpson β: Bray-Curtis
Ye et al. [48]	China	DSM-IV HDRS	27.9(2.8)	P: 26 HC: 28	P: 26.0 HC: 26.0	P: 80.8 HC: 75.0	P: 19.8 HC: 21.6	Nr	Nr	No	16S rRNA V3-V4	α: Chao1, Shannon β: UniFrac (unweighted)
Yuan et al. [49]	China	PHQ-9	12.5(5.4)	P: 49 HC: 62	P: 36.9 HC: 41.6	P: 59.2 HC: 58.1	P: 22.1 HC: 23.3	Yes	Yes	Nr	16S rRNA V3-V4	α: Shannon, PD β: not measured
Zhang et al. [50]	China	ICD-10 HDRS	14.1(3.0)	P: 36 HC: 45	P: 36.8 HC: 39.3	P: 41.7 HC: 57.8	P: 24.5 HC: 23.9	Yes	Nr	No	16S rRNA V4-V5	α: Chao, ACE, Shannon, Simpson β: UniFrac (weighted & unweighted), Bray-Curtis, Jaccard
Zhang et al. [96]	China	HDRS	19.5(0.8)	P: 40 HC: 30	P: 40.9 HC: 44.2	P: 73.7 HC: 69.6	Nr	Nr	Nr	No	16S rRNA V4	α:Chao, ACE, Observed sp. β: Bray-Curtis
Zhao et al. [51]	China	DSM-5 HDRS	24.8(3.1)	P: 24 HC: 26	P: 30.0 HC: 31.3	P: 70.8 HC: 69.2	Nr	Nr	No	No	Nr	α: not measured β: not measured
Zheng et al. [19]	China	DSM-IV-TR HDRS	P: 22.8(4.4)	P: 58 HC: 63	P: 40.6 HC: 41.8	P: 62.1 HC: 63.5	P: 22.0 HC: 22.6	Yes	Nr	Yes	16S rRNA V3-V5	α: Observed sp., Shannon, Simpson, PD β: UniFrac (weighted & unweighted)
Zheng et al. [52]	China	DSM-IV HDRS	22.9	P: 165 HC: 217	P: 29.3 HC: 30.8	P: 64.2 HC: 56.2	P: 22.3 HC: 22.5	Nr	Nr	Yes	16S rRNA V3-V4	α:Chao, ACE, Shannon, Inv. Simpson β:PLS-DA
Zheng et al. [53]	China	ICD-10 HDRS	20.2(7.9)	P: 30 HC: 30	P: 30.8 HC: 33.4	P: 60.0 HC: 56.7	P: 21.5 HC: 22.9	Nr	Nr	No	16S rRNA Nr	α: Chao1, ACE, Shannon, Simpson, Sobe β: not measured
Zhou et al. [54]	China	DSM-IV HDRS	13.5(3.5)	P: 39 HC: 18	P: 33.6 HC: 32.6	P: 100.0 HC: 100.0	P: 21.5 HC: 20.9	Nr	Nr	No	16S rRNA V4	α:Observed sp., Shannon, PD, Evenness β:UniFrac (weighted)
Zhou et al. [97]	China	ICD-10 SDS	76.3(8.5)	P: 70 HC: 101	P: 13.7 HC: 13.5	P: 62.2 HC: 53.4	P: 19.6 HC: 19.9	Yes	No	Yes	16S rRNA V3-V4	α:Chao1, ACE, Shannon, Sobs β:UniFrac (weighted)

*BMI* body mass index, *P* patient, *HC* healthy control, *ICD* International Classification of Diseases, *DSM* Diagnostic and Statistical Manual of Mental Disorders, *MINI* Mini-International Neuropsychiatric Interview, *GP* general practitioner, *CES-D* Center for Epidemiologic Studies Rating Scale for Depression, *ACE* abundance-based coverage estimator. *RT-qPCR* real time quantitative polymerase chain reaction, *PD* phylogenetic diversity, *PLS*-*DA* partial least squares discriminant analysis, *OPLS-DA* orthogonal projections to latent structures discriminant analysis, *n* number, *Nr* not reported, *HDRS* Hamilton Depression Rating Scale, *PHQ-9* Patient Health Questionnaire-9.

### Characteristics of included studies

The selected articles consisted of 44 case-control studies [19, 22–54, 88–97]. The total number of participants was 4883 (2091 [42.8%] in the depressive group and 2792 in healthy groups). The mean number of included patients in the studies was 48 (range 7–167), and the mean number of healthy controls was 63 (range 10 to 1071). Thirty-two studies (72.7%) were conducted in East Asia (China [19, 24, 27, 28, 30, 32–36, 38, 41, 42, 47–54, 89, 90, 93, 94, 96, 97], Japan [23], Korea [91], and Taiwan [29, 88, 95]), 12 (27.3%) in westernized populations (USA [37, 39, 43–45], Australia [26], Norway [40], Spain [25], Italy [31], Ireland [22], Russia [92], and Belgium [46]), grouped according to typical diet and lifestyle. Studies were similar in exclusion criteria, however, few attempted to minimize dietary changes or control dietary intake (7 [29, 31, 38, 47, 49, 91, 95] of 44 [15.9%]). The diagnosis of depression was assessed using the MINI, the DSM, and the ICD-10, while one study [96] assessed using only the HDRS, one [46] using GP-reported, one [91] using CES-D and one [49] using PHQ-9. In addition, 28 studies (63.6%) used the HAMD to examine the severity of depression symptoms [19, 22–25, 27, 28, 30, 33–36, 41, 42, 47, 48, 50–54, 89, 90, 92–96]. Only seven of all studies did not report BMI of participants [26, 37, 44, 45, 51, 93, 96]. Smoking status (17 [19, 22, 25–29, 31, 33, 35, 37, 42, 49, 50, 89, 93, 97] of 44 [38.6%]) and alcohol consumption (17 [22, 24–28, 30, 31, 39, 42, 45, 49, 51, 89, 93, 94, 97] of 44 [38.6%]) was reported. Use of psychiatric medication also varied substantially, with 18 [24, 27, 30, 32, 36, 38, 42, 48, 50, 51, 53, 54, 89, 90, 92–94, 96] of 44 studies (40.9%) conducted in medication-free or drug-naive groups, 21 [19, 22, 23, 25, 26, 28, 29, 31, 33–35, 37, 39, 41, 44–47, 52, 88, 97] of 44(47.7%) in groups undergoing treatment and the remainder not controlling this, resulting in anywhere between 10.9% and 100% of patients taking medication. Composition analysis (Table 1) varied widely, with 16 S ribosomal RNA sequencing being most common (35 [19, 22, 24–26, 29–33, 35–40, 42, 44–46, 48–50, 52–54, 88–91, 93–97] of 44 studies [79.5%]) followed by 1 study (2.3%) [23] using real-time quantitative polymerase chain reaction(RT-qPCR),1 study(2.3%) [28] using metaproteomics, 5 (11.4%) [34, 41, 43, 47, 92] using shotgun metagenomics and 1 study(2.8%) [27] using not only 16 S ribosomal RNA but also shotgun metagenomics. Methodology of stool processing (Table S2) showed that the far most common storage temperature was −80° (36 [19, 22, 25, 26, 28–34, 36–45, 48–51, 53, 54, 88–90, 92–97] of 44[81.8%]).

### Alpha diversity

A total of 35 case-control studies in depressive disorder examined alpha diversity indices (Table S3) [19, 22, 24–27, 29, 30, 32–34, 36–38, 40–42, 45, 47–50, 52–54, 88–97]. Because four studies did not provide relevant data [26, 49, 50, 53], thirty-one studies were included in meta-analyses [19, 22, 24, 25, 27, 29, 30, 32–34, 36–38, 40–42, 45, 47, 48, 52, 54, 88–97]. Eleven indices were used to assess alpha diversity, including estimates of richness (observed species, Chao1, abundance coverage estimator [ACE]), biodiversity (Shannon, Simpson, inverse Simpson, Pielou’s, Fisher, Faith’s phylogenetic diversity), and 3 newly developed indices [41, 53, 97]. The most widely used were observed species index, Chao1 index, ACE index, Shannon index, Simpson index and phylogenetic diversity index. The observed species index, Chao1 index and ACE index reflected the abundance of the community. The Shannon index, Simpson index, and phylogenetic diversity index reflected the diversity of the community.

**Fig. 2:**
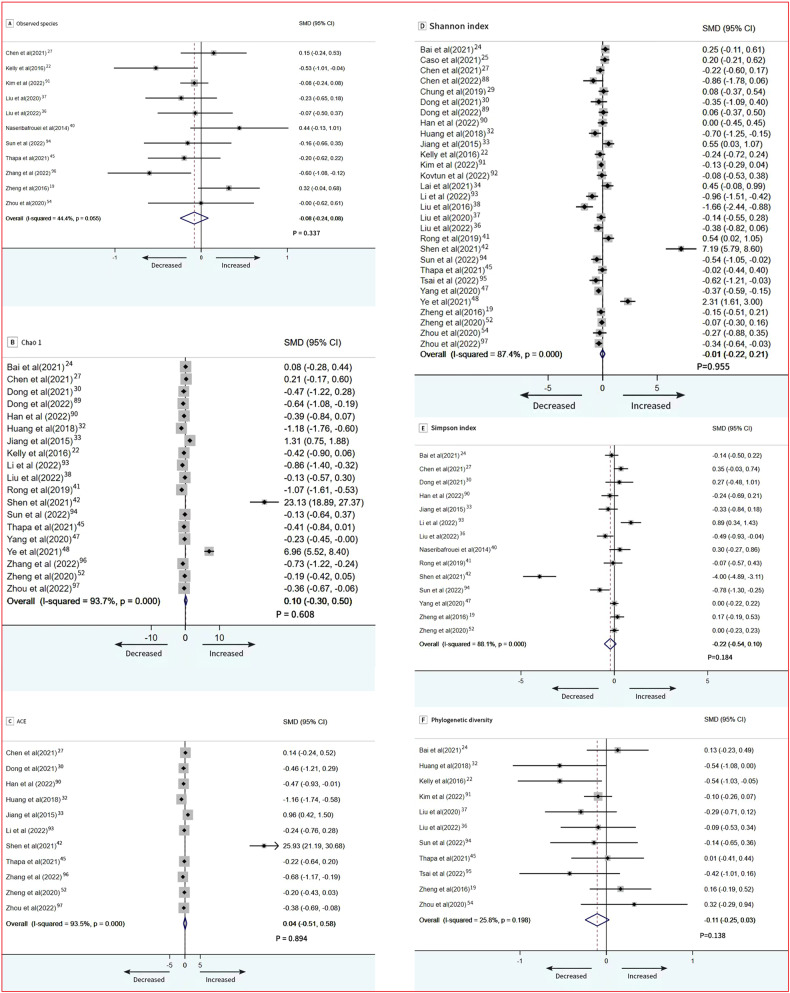
Forest plots of alpha diversity in the gut microbiota of patients with depressive disorder compared with healthy controls. **A** Observed; **B** Chao1; **C** ACE; **D** Shannon index; **E** Simpson index; **F** Phylogenetic diversity.

Regarding richness, 11 studies provided data on observed species in patients (*n* = 650) vs controls (*n* = 1421). The pooled estimate showed no significant difference between groups (SMD = −0.08; 95% CI, −0.24 to 0. 08; *P* = 0.337) and no significant heterogeneity (Fig. 2A) [19, 22, 27, 36, 37, 40, 45, 54, 91, 94, 96]. Chao1 data were provided by 19 studies (1045 patients and 930 controls). There was a no significant difference between groups (SMD = 0.10; 95% CI, −0.30 to 0.50; *P* = 0.608), with high heterogeneity (*I*^2^ = 93.7%) (Fig. 2B) [22, 24, 27, 30, 32, 33, 36, 41, 42, 45, 47, 48, 52, 89, 90, 93, 94, 96, 97]. Eleven studies reported data on ACE in patients (*n* = 604) vs controls (*n* = 524).There was a no significant difference between groups (SMD = 0.04; 95% CI, −0.51 to 0.58; *P* = 0.894), with high heterogeneity (*I*^2^ = 93.5%) (Fig. 2C) [27, 30, 32, 33, 42, 45, 52, 90, 93, 96, 97].

Regarding diversity, random-effects between-group meta-analysis showed that there was no significant difference in patients with depressive disorder compared with healthy controls on Shannon index whose data was provided by 29 studies (1506 patients, 2293 controls; SMD = −0.22; 95% CI, −0.22 to 0.21; *P* = 0.955) with high heterogeneity (*I*^2^ = 87.4%) (Fig. 2D) [19, 22, 24, 25, 27, 29, 30, 32–34, 36–38, 41, 42, 45, 47, 48, 52, 54, 88–95, 97]. Simpson index data were provided by 14 studies (770 patients, 735 controls). There was no significant difference between groups (SMD = −0.22; 95% CI, −0.54 to 0.10; *P* = 0.184), with high heterogeneity (*I*^2^ = 88.1%; Fig. 2E) [19, 24, 27, 30, 33, 36, 40–42, 47, 52, 90, 93, 94]. Finally, 11 studies provided phylogenetic diversity data in patients (*n* = 634) vs controls (*n* = 1431). The pooled estimate showed no significant difference between groups (SMD = −0.11; 95% CI, −0.25 to 0.03; *P* = 0.138) and no significant heterogeneity (Fig. 2F) [19, 22, 24, 32, 36, 37, 45, 54, 91, 94, 95].

**Fig. 3:**
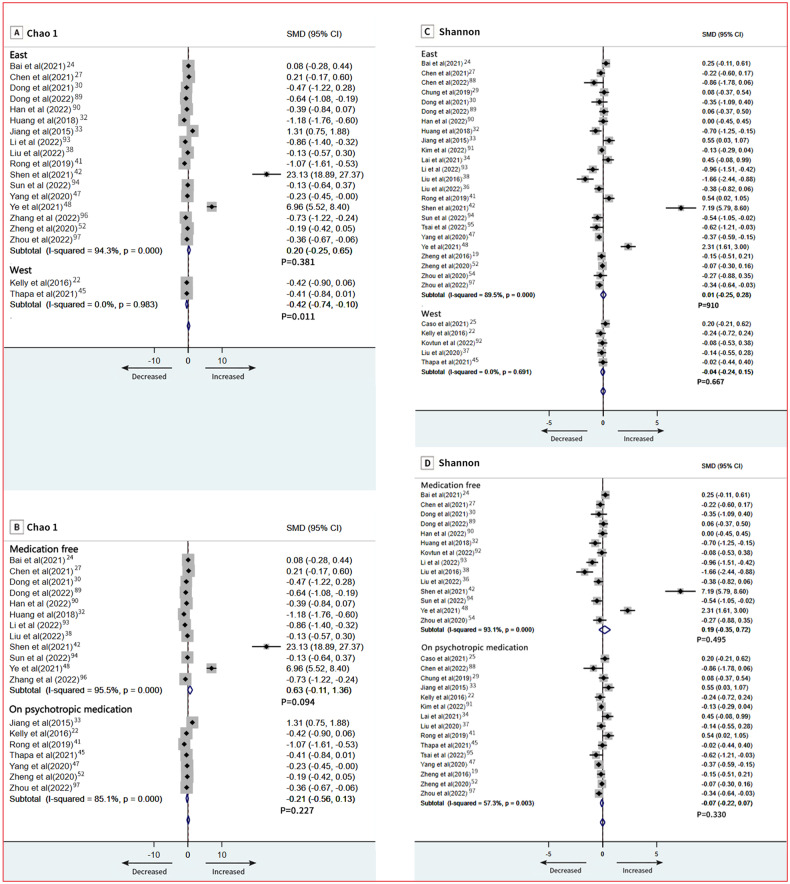
Subgroup meta-analysis of Chao 1 index and Shannon index. **A** Chao1; **B** Chao1; **C** Shannon; **D** Shannon.

In subgroup analyses of patients with depression by regional variations(east/west), Chao1 was different in the depression group. Patients who were in the West had fewer number of species (SMD = −0.42; 95% CI, −0.74 to −0.10; *P* = 0.011; Fig. 3A). Subgroup meta-analyses using regional variations(east/west) and psychotropic medication as predictor variables were not significantly different as assessed by Shannon index (Fig. 3C, D).

In our investigation of other sources of heterogeneity using meta-regression analyses, meta-regression models, severity of depressive symptoms as assessed by HDRS, was not related to the magnitude of the effect size, indicating no association between severity of symptoms and alpha diversity (Chao1 and Shannon) (Fig. S1A, D). Additionally, we found no relation between age, sex, and BMI in depression and alpha diversity (Chao1 and Shannon; Table S4).

### Beta diversity

Of the 44 case-control studies, 34 studies analyzed beta diversity, using a variety of measures (Table S5) [19, 22, 24–27, 29, 30, 32–37, 39, 41, 42, 44, 45, 47, 48, 50–52, 54, 88–92, 94–97]. Consistent nonsignificant differences were reported by 12 studies, and a further 3 reported conflicting results between the measures used. Nineteen studies found significant differences in beta diversity between patients with depressive disorder and controls (Table S5). Mason et al. [39] found no difference between participants with depression and controls when stratified by diagnosis, but hierarchical clustering of beta diversity identified two participant groups associated with anhedonia scores derived from self-report questionnaires (weighted UniFrac). These findings suggest there is reliable evidence for differences in the phylogenetic relationship in depressive disorder compared with controls, however, method of measurement and method of patient classification (symptom vs diagnosis based) may affect findings.

### Summary of representative taxa in the observational trials

**Fig. 4:**
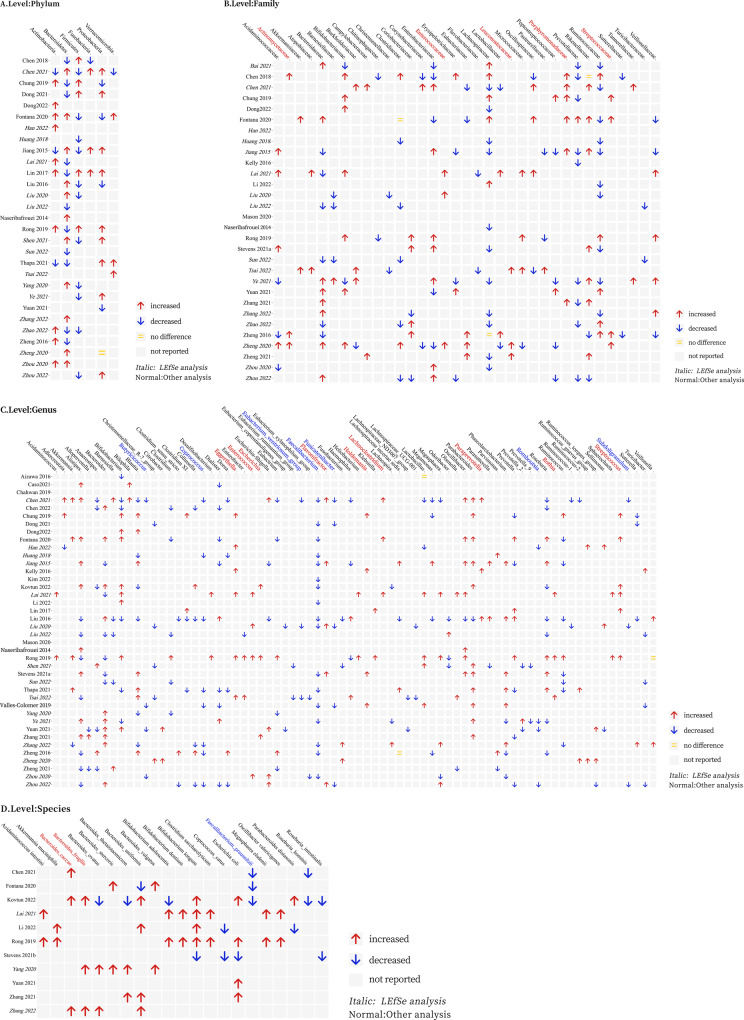
Changes in relative abundance of microbial taxa reported by at least 2 studies. **A** Level Phylum; **B** Level Family; **C** Level Genus; **D** Level Species.

Of the 44 studies which analyzed the gut microbiota in depressive disorder, 19 studies(43.2%) presented taxa specific results based on a Linear discriminant analysis effect size (LEfSe) analysis and all reported findings with a Linear discriminant analysis (LDA) scoreå 2 or ≤2 [24, 27, 32–34, 36, 37, 42, 47, 48, 50–52, 54, 90, 94–97]. Most studies identified significant differences between patients and controls at phylum, family, or genus levels. Owing to the significant likelihood of false [62], we summarized findings for each taxon reported in at least 2 studies and labeled those increased, decreased, or not changed (Figs. 4 and S2).

**Fig. 5:**
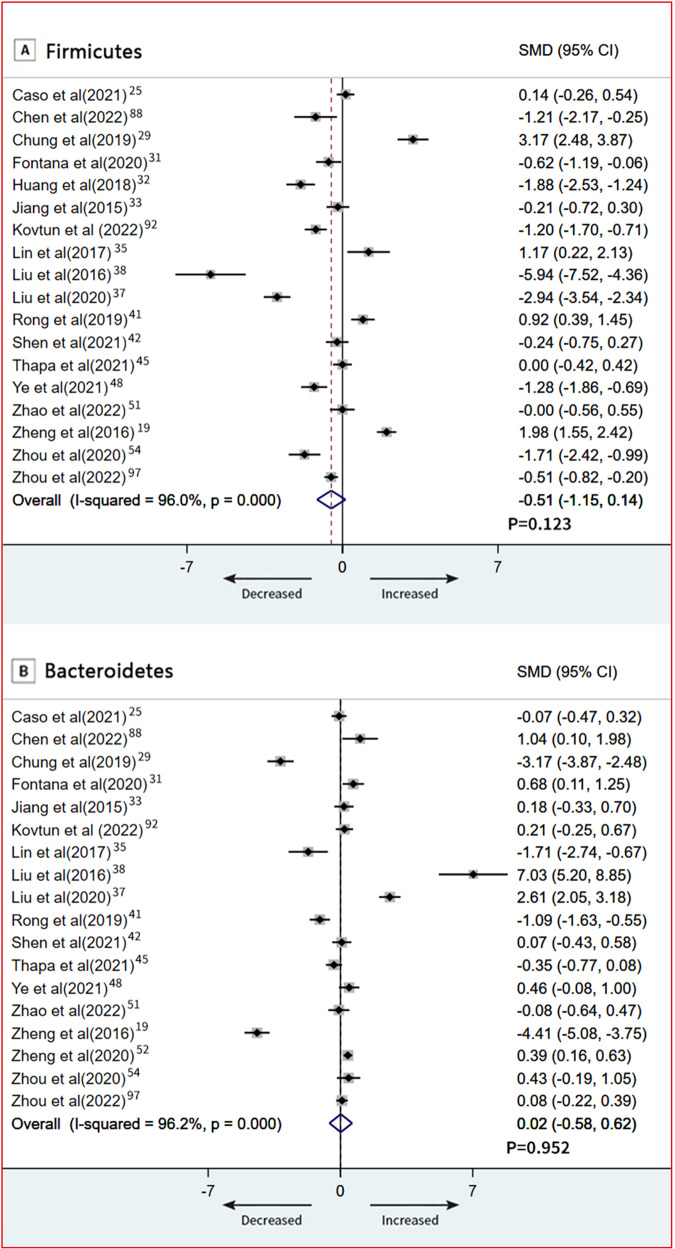
Forest plots of phylum level in the gut microbiota of patients with depressive disorder compared with healthy controls. **A** Firmicutes; **B** Bacteroidetes.

**Fig. 6:**
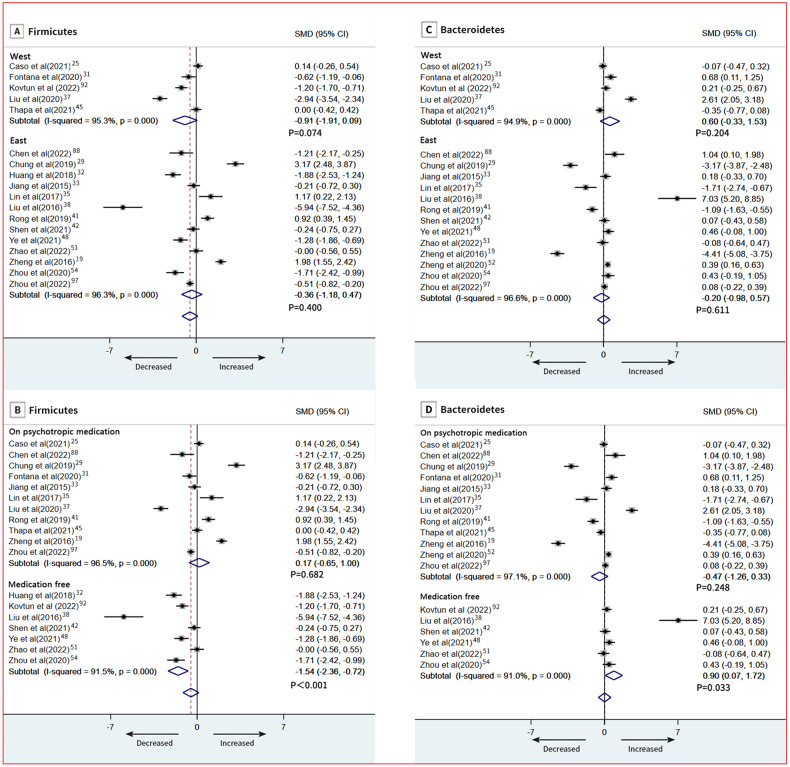
Subgroup meta-analysis of Firmicutes and Bacteroidetes. **A** Firmicutes; **B** Firmicutes; **C** Bacteroidetes; **D** Bacteroidetes.

At the phylum level, 18 studies provided data on Firmicutes in patients (*n* = 665) vs controls (*n* = 609) [19, 25, 29, 31–33, 35, 37, 38, 41, 42, 45, 48, 51, 54, 88, 92, 97]. The pooled estimate showed no significant difference between groups (SMD = −0.51; 95% CI, −1.15 to 0.14; *P* = 0.123), with high heterogeneity (*I*^2^ = 96.0%; Fig. 5A). Bacteroidetes data were provided by 18 studies (760 patients and 753 controls) [19, 25, 29, 31, 33, 35, 37, 38, 41, 42, 45, 48, 51, 52, 54, 88, 92, 97]. There was a no significant difference between groups (SMD = 0.02; 95% CI, −0.58 to 0.62; *P* = 0.952; Fig. 5B). Results of our subgroup meta-analyses for psychotropic medication showed that Firmicutes remained significantly different in patients with depressive disorder who were medication free (SMD = −1.54; 95% CI, −2.36 to −0.72; *P* = 0.033; Fig. 6B), and Bacteroidetes also remained significantly different in patients with depressive disorder who were medication free (SMD = 0.90; 95% CI, 0.07 to 1.72; *P* < 0.001; Fig. 6D). Meta-regression models, severity of depressive symptoms as assessed by HDRS, was not related to the magnitude of the effect size, indicating no association between severity of symptoms and phylum level (Firmicutes and Bacteroidetes) (Fig. S1C, D). Additionally, we found no relation between age, sex, and BMI in depression and phylum level (Firmicutes and Bacteroidetes; Table S4). An overview of the family level is provided in Fig. 4A.

At the family level, over two studies consistently found that the abundance of *Actinomycetaceae*, *Enterococcaceae, Leuconostocaceae, Porphyromonadaceae*, and *Streptococcaceae* were higher in depression relative to controls. *Prevotellaceae* was lower in depressive disorders reported by nine studies [22, 24, 28, 29, 33, 48, 50, 51, 97] and only one [31] reported higher abundance. An overview of the family level is provided in Fig. 4B.

At the genus level, at least 3 studies reported that depleted levels of *Butyricicoccus, Coprococcus, Eubacterium_ventriosum_group, Faecalibacterium, Fusicatenibacter, Romboutsia, Subdoligranulum* and enriched *Eggerthella, Enterococcus, Escherichia, Flavonifractor, Holdemania, Lachnoclostridium, Paraprevotella, Rothia*, and *Streptococcus* were consistently shared in depressive disorder. *Megamonas* was lower in depression in four studies [19, 27, 29, 38], although the opposite was observed in one study [33]. A higher abundance of *Oscillibacter* and *Parabacteroides* was reported in depression in all the reported studies, except one study [38] reported the opposite result. Finally, lower levels of *Odoribacter* were observed in three studies in depression, [38, 41, 42] although the opposite was observed in one study [36]. An overview of genus level is provided in Fig. 4C.

The vast majority of included studies were based on 16S rRNA gene sequencing, and consequently, they do not provide enough taxonomic resolution to report results at the species level. Nevertheless, at least 3 case-control studies independently reported several overlapping findings, including a higher relative abundance of *Bacteroides_caccae*, *Bacteroides_fragilis*, and a lower relative abundance of *Faecalibacterium_prausnitzii* in depressive disorders relative to controls. An overview of the species level is provided in Fig. 4D.

We explored the association of study region (east/west) with microbial alterations. Owing to the imbalanced availability of studies by region (70.5% included studies were largely investigated in the east), this analysis should be considered preliminary. Clustering according to region identified several taxa that were altered only in over two studies from Eastern countries: *Adlercreutzia*, *Alloprevotella*, *Barnesiella*, Clostridium_sensu_stricto, *Enterobacter*, *Escherichia-Shigella*, *Fusobacterium*, *Haemophilus*, *Megamonas*, *Megasphaera*, *Odoribacter*, *Olsenella*, *Parasutterella*, *Parvimonas*, *Prevotella_2*, *Ruminococcus_torques_group*, *Sutterella*, and *Veillonella* were not consistent; *Rothia* and *Sphaerochaeta* were increased; *Romboutsia* and *Eubacterium_ventriosum_group* were decreased. These differences were driven entirely by studies from China, highlighting the need to distinguish the Chinese microbiome from other East Asian nations as more evidence becomes available.

We also investigated effects of psychiatric medication on microbiota composition. We found that increased in the family *Actinomycetaceae*, *Enterococcaceae*, *Streptococcaceae* and the genera *Holdemania, Rothia*, and *Streptococcus* were only reported in medicated groups, while *Dialister* was decreased in medicated and increased in medication-free groups. These results indicated psychiatric medication can affect microbiota composition.

Results from the sensitivity analyses, and publication bias are shown in the appendix (Figs. S3 and S4).

## Discussion

We assessed gut microbiota alterations of depressive disorder with the aim of evaluating the reproducibility and specificity of potential gut microbial biomarkers. The systematic review revealed substantial methodological differences between the included studies both regarding demography and storage of fecal samples with differences in analyses methods and reporting of study findings. It reports that there were no significant differences in patients with depressive disorder on alpha diversity indices, Firmicutes and Bacteroidetes compared with healthy controls. In subgroup analyses with regional variations(east/west) as a predictor, Patients who were in the West had a lower Chao1 level (moderate effect size). Subgroup meta-analysis showed that Firmicutes level was decreased in patients with depressive disorder who were medication free (large effect size), but Bacteroidetes level was increased (large effect size). Subgroup meta-analyses using regional variations(east/west) and psychotropic medication as predictor variables were not significantly different as assessed by Shannon index. In the meta-regression analysis, six variables—regional variations (east/west), use of psychotropic medication, age, sex, BMI, and HDRS—cannot explained the 100% heterogeneity of the studies assessing by Chao1, Shannon index, Firmicutes and Bacteroidetes; which would suggest the presence of unmeasured moderators. All studies reviewed found significant differences in taxa between depression and control groups. However, there was minimal consensus regarding either microbial diversity or relative abundance or directionality of differences in taxa associated with depressive disorders. The most consistent changes in the microbiota were a higher relative abundance of *Eggerthella, Enterococcus, Escherichia, Flavonifractor, Holdemania, Lachnoclostridium, Paraprevotella, Rothia*, *Streptococcus* and lower for *Butyricicoccus, Coprococcus, Faecalibacterium, Eubacterium_ventriosum_group, Fusicatenibacter, Romboutsia, Subdoligranulum* at genus level.

Lower diversity in patients relative to controls has been reported in some diseases and mental health disorders [98–102], however, our quantitative synthesis of included studies revealed no significant differences in the SMDs of the observed species, Chao1, ACE, phylogenetic diversity, Shannon and Simpson Index between patients with depressive disorder and healthy controls. The use of alpha diversity indices in biomedical research stems from the assumption that “higher diversity is somehow more meritorious ecologically”, and that diversity of species provides a proxy for microbial function and stability that is assumed to be favorable for the host [64]. Nevertheless, the present review reveals that this conclusion is, at present, unfounded in depression. Regarding beta diversity (between samples), patients with depressive disorder consistently clustered differently from controls. However, we could not conduct a meta-analysis due to insufficient data.it is yet unknown whether depressive disorder cluster differently from other psychiatric disorders, thus questioning the suitability of diversity measures as biomarkers. Moreover, existing multivariate analyses which compare beta diversity between groups often involve dichotomization based on the absence or presence of a condition (PCoA/PERMANOVA). Research in depressive disorder thus encounters several methodological challenges not faced by conditions with defined biomarkers, including the disparate methods by which clinical groups are defined (e.g., self-reported symptom levels, psychiatrist diagnosis, clinical interviews). Accordingly, incorporation of bias due to nosology requires consideration in future research.

Notwithstanding Firmicutes and Bacteroidetes are observed to alter in the human studies related to the gut microbiota and depression [84], however, our quantitative synthesis of included studies revealed no significant differences in the SMDs of the Firmicutes and Bacteroidetes between patients with depressive disorder and healthy controls.

Although diversity and phylum level findings were nonsignificant differences, specific bacterial taxa were implicated in studies that compared the gut microbiota of depression groups relative to controls. Among the most consistent findings was a lower abundance of *Butyricicoccus, Coprococcus, Faecalibacterium, Fusicatenibacter, Eubacterium_ventriosum_group, Romboutsia, Subdoligranulum*, as well as a higher abundance of *Eggerthella, Enterococcus, Escherichia, Flavonifractor, Holdemania, Lachnoclostridium, Paraprevotella, Rothia*, and *Streptococcus* in participants with depressive disorder relative to controls. Several mechanisms by which these taxa may be associated with depression, focusing on increased proinflammatory communication and anti-inflammatory communication via the gut-brain axis.

Several taxa reported to have a higher relative abundance in clinical depression were associated with gastrointestinal inflammation (i.e., *Enterococcus, Eggerthella*) [103–105]. Inflammation has been widely suggested as a contributor to the pathogenesis of depressive disorders [106, 107]. Consistently, growing shreds of evidence of continuous low-level immune-inflammatory reaction also cannot be ignored and the source of this immune inflammation reaction is probably related to the disorder of gut microbiota [19, 22, 108, 109]. The potential for microbiota-mediated inflammation in depression is not only indicated by the increase in inflammation-associated microbial members but may be further exacerbated by a loss of species that secrete anti-inflammatory metabolic products. Our review revealed a reduction of *Faecalibacterium*, *Coprococcus*, and *Butyricicoccus* which have been demonstrated to secret anti-inflammatory the short-chain fatty acid (SCFAs) particularly butyrate in depression groups relative to controls [110–112], and *Sutterella, Megamonas* which can produce acetate and propionate [113]. For *Oscillibacter*, which was observed increase, the type strain of this genus has valeric acid as its main metabolic end product. [114] Gut microbiota can also regulate brain function by influencing tryptophan metabolism [115] Some bacteria, such as *Enterococcus, Lactobacillu* and *Oscillibacter*, which encode Trp synthase genes, are found to increase mainly in included studies. Trp and 5-HTP (precursor of 5-HT) can pass through the blood-brain barrier (BBB) and become the precursors of 5-HT in the brain.

Many of the included studies were conducted in Asia regions. Geographic/ethnic dietary differences may be expected to affect the gut microbiome directly [74], differences in microbial composition vary considerably by geographical location [69]. Our study revealed that patients who were West had a lower Chao1 level, but the result was not observed in Shannon index, indicating regional variations(east/west) may only influence number of species of intestinal flora, not affect the evenness of gut microbiota. Given the small number of studies in the subgroup meta-analysis, this limits the interpretation and generalization of findings, therefore, additional studies are needed for a definitive conclusion on this issue. Consistently, a study of regionally heterogeneous participants did find a signal for mental illness status that was reproducible among subsets by region, although the mental illness status was determined by self-report and covered several conditions in addition to depression [69]. Almost all studies did not consider the effects of diet, some of the selected studies collected the diet information by interview [38], questionnaire [29, 47, 49, 91, 95], and dietary records [31]. It is well known that diet would potentially affect the distribution of microbiota and their function [116]. Consumption of a high-fat and animal protein diet has also been associated with elevated Actinobacteria [117], and the majority of studies that reported differences in Actinobacteria did not control for diet [19, 27, 28, 33–35, 41, 45, 51, 54, 89, 90]. Further investigations which adequately model dietary intake are required to disentangle whether associations are driven by dietary intake or independently associated with depression.

There was evidence in the literature that psychotropic medications may impact the gut microbiome, for example, atypical antipsychotics are associated with altered gut microbiota in rodents [70–72]. In a study of bipolar adults, significant changes in the abundance of three genera were identified between those taking vs. not psychotropic medications [73]. In our study, Subgroup meta-analysis showed Firmicutes level was decreased in patients with depressive disorder who were medication free, but Bacteroidetes level was increased, indicating psychotropic medications may influence gut microbiota. Accordingly, incorporation of bias due to psychotropic medications requires consideration in future research.

The selected studies had a wide range of ages. In general, there are differences in the distribution of microbiota according to age [118, 119]. For example, Firmicutes is the dominant taxa during the neonatal period, but Actinobacteria and Proteobacteria are about to increase in three to six months [120]. While in adults, Vemuri et al. [121] reported that Bacteroidetes and Firmicutes were the dominant taxa. Meanwhile, compared to younger individuals, the abundance of Bacteroidetes is significantly higher in frailer older individuals [122]. Similarly, Chen et al. (2020) identified 6 and 25 differentially abundant bacterial taxa responsible for the differences between MDD patients (young and middle-aged, respectively) and their respective HCs [123]. However, we found no relation between age in depression and Chao1, Shannon index, Firmicutes and Bacteroidetes. Given the small number of studies in the meta-regression, this limits the interpretation and generalization of findings, therefore, additional studies are needed for a definitive conclusion on this issue.

There is clear sex difference in the prevalence of depressive disorder, both being more common in females than males [1]. Sex differences in gut microbiota composition have also been suggested to underlie susceptibility to gut-microbiota-mediated conditions in females [124]. In the few studies that examined males and females separately, significant differences were observed, sometimes with effects in opposite directions (e.g., *Lachnospiraceae*, *Coriobacteriaceae*, and *Erysipelotrichaceae incertae sedis*) [125]. However, we found no relation between sex in depression and Chao1, Shannon index, Firmicutes, and Bacteroidetes.

In the meta-regressions we found no association between depressive symptoms, BMI in depression and Chao1,Shannon index, Firmicutes, and Bacteroidetes.

Based on our narrative synthesis heterogeneity of studies was visible and studies reporting no significant results were prominent, yet tend to not report sufficient data for inclusion in meta-regressions, resulting in a bias in the meta-regressions on significant effects. These limitations may have resulted in false-negative result in our meta- regressions, and results should thus be interpreted with caution. Therefore, additional studies are needed for a definitive conclusion on the sources of heterogeneity.

The use of metaproteomics, shotgun metagenomic, and RT-qPCR affects comparability with the other studies, which used 16 S RNA quantification. Furthermore, in contrast to most of the studies, three studies limited their search of the gut microbiome a priori to specific taxa: Firmicutes and Bacteroidetes [28, 32] or Bifidobacterium and Lactobacillus [23]. Even though most studies used high throughput sequencing of 16 S rRNA, the analysis methods involved different variable regions, different pipelines, different databases, and different cut-offs, which may each influence results to varying degrees. Moreover, analytic methods differed widely among the studies. Statistical methodology for microbiome analysis has not been standardized across the field, and many approaches have been noted to be prone to high false discovery rates [126].

An important strength of our systematic review was the search strategy, since we have used a range of databases and made an exhaustive effort to acquire data. Our study has some inherent limitations. First, the meta-regressions might have failed to achieve statistical significance because of a lack of power in these specific analyses, thus giving a false-negative result. Second, our meta-analyses on alpha diversity indices and phylum level in patients with depressive disorder compared with healthy controls provided us with pooled results originating from cross-sectional studies, and we therefore cannot draw any conclusions on causality. Third, waist circumference is more accurate than BMI for assessment of visceral adiposity. Since most studies included in our analysis did not provide data on waist circumference, we used BMI as a surrogate for visceral adiposity. Finally, any meta-analysis is dependent on the quality of the analyzed studies, and our results need to be verified by studies specifically designed to test the points we raised. Finally, poor quality studies were included and included studies have publication bias. The limitations should be considered in future syntheses. Many other approaches to the complex field of the gut and brain than case-control studies can be carried out and it is yet far too early to conclude that the gut microbiota is different, and if it is the matter of causality remains to be addressed. Studies with more participants (especially of psychosis and major depressive disorder) are needed, and these studies should focus not only on the gut microbial composition but also on the function, carefully taking confounders into account, thereby making it possible to analyze data in a wider perspective than to date.

## Conclusion

This systematic review and meta-analysis found that psychotropic medication and dietary habit can influence microbiota. There is reliable evidence for differences in the phylogenetic relationship in depressive disorder compared with controls; however, method of measurement and method of patient classification (symptom vs diagnosis based) may affect findings. Depressive disorder is characterized by a reduction of anti-inflammatory butyrate-producing bacteria, while pro-inflammatory genera are enriched. Future research should assess confounders and examine microorganism function to prevent unmerited claims of disorder specificity of gut microbial biomarkers.

### Supplementary information

Supplementary materials

## Data Availability

The data that support the findings of this study have been included in the main text and Supplementary Information. All other relevant data supporting the findings of this study are available from the corresponding authors upon request.

## References

[1] Sampson L, Ettman CK, Galea S (2020). Urbanization, urbanicity, and depression: a review of the recent global literature. Curr Opin Psychiatry.

[2] Doran CM, Kinchin I (2019). A review of the economic impact of mental illness. Aust health Rev: Publ Aust Hosp Assoc.

[3] Jiang HY, Pan LY, Zhang X, Zhang Z, Zhou YY, Ruan B (2020). Altered gut bacterial-fungal interkingdom networks in patients with current depressive episode. Brain Behav.

[4] Pan R, Zhang X, Gao J, Yi W, Wei Q, Su H (2020). Analysis of the diversity of intestinal microbiome and its potential value as a biomarker in patients with schizophrenia: a cohort study. Psychiatry Res.

[5] Jiang HY, Zhang X, Yu ZH, Zhang Z, Deng M, Zhao JH (2018). Altered gut microbiota profile in patients with generalized anxiety disorder. J Psychiatr Res.

[6] Hemmings SMJ, Malan-Müller S, van den Heuvel LL, Demmitt BA, Stanislawski MA, Smith DG (2017). The Microbiome In Posttraumatic Stress Disorder And Trauma-exposed Controls: An Exploratory Study. Psychosom Med.

[7] Monteleone AM, Troisi J, Fasano A, Dalle Grave R, Marciello F, Serena G (2021). Multi-omics data integration in anorexia nervosa patients before and after weight regain: A microbiome-metabolomics investigation. Clin Nutr.

[8] Turna J, Grosman Kaplan K, Anglin R, Patterson B, Soreni N, Bercik P (2020). The gut microbiome and inflammation in obsessive-compulsive disorder patients compared to age- and sex-matched controls: a pilot study. Acta Psychiatr Scand.

[9] Kennedy PJ, Cryan JF, Dinan TG, Clarke G (2017). Kynurenine pathway metabolism and the microbiota-gut-brain axis. Neuropharmacology.

[10] Waclawiková B, El Aidy S. Role of microbiota and tryptophan metabolites in the remote effect of intestinal inflammation on brain and depression. Pharmaceuticals 2018;11:63. 10.3390/ph11030063 PMC6160932 29941795

[11] Bravo JA, Forsythe P, Chew MV, Escaravage E, Savignac HM, Dinan TG (2011). Ingestion of Lactobacillus strain regulates emotional behavior and central GABA receptor expression in a mouse via the vagus nerve. Proc Natl Acad Sci USA.

[12] Bastiaanssen TFS, Cowan CSM, Claesson MJ, Dinan TG, Cryan JF (2019). Making sense of the microbiome in psychiatry. Int J Neuropsychopharmacol.

[13] Forsythe P, Kunze WA, Bienenstock J (2012). On communication between gut microbes and the brain. Curr Opin Gastroenterol.

[14] Dowlati Y, Herrmann N, Swardfager W, Liu H, Sham L, Reim EK (2010). A meta-analysis of cytokines in major depression. Biol Psychiatry.

[15] Severance EG, Prandovszky E, Castiglione J, Yolken RH (2015). Gastroenterology issues in schizophrenia: why the gut matters. Curr Psychiatry Rep.

[16] Arneth BM (2018). Gut-brain axis biochemical signalling from the gastrointestinal tract to the central nervous system: gut dysbiosis and altered brain function. Postgrad Med J.

[17] Mayer EA, Knight R, Mazmanian SK, Cryan JF, Tillisch K (2014). Gut microbes and the brain: paradigm shift in neuroscience. J Neurosci.

[18] Gacias M, Gaspari S, Santos PM, Tamburini S, Andrade M, Zhang F et al. Microbiota-driven transcriptional changes in prefrontal cortex override genetic differences in social behavior. Elife 2016;5:e13442. 10.7554/eLife.13442 PMC4880443 27097105

[19] Zheng P, Zeng B, Zhou C, Liu M, Fang Z, Xu X (2016). Gut microbiome remodeling induces depressive-like behaviors through a pathway mediated by the host’s metabolism. Mol Psychiatry.

[20] Doolin K, Allers KA, Pleiner S, Liesener A, Farrell C, Tozzi L (2018). Altered tryptophan catabolite concentrations in major depressive disorder and associated changes in hippocampal subfield volumes. Psychoneuroendocrinology.

[21] Knudsen JK, Michaelsen TY, Bundgaard-Nielsen C, Nielsen RE, Hjerrild S, Leutscher P (2021). Faecal microbiota transplantation from patients with depression or healthy individuals into rats modulates mood-related behaviour. Sci Rep.

[22] Kelly JR, Borre Y, O’ Brien C, Patterson E, El Aidy S, Deane J (2016). Transferring the blues: depression-associated gut microbiota induces neurobehavioural changes in the rat. J Psychiatr Res.

[23] Aizawa E, Tsuji H, Asahara T, Takahashi T, Teraishi T, Yoshida S (2016). Possible association of Bifidobacterium and Lactobacillus in the gut microbiota of patients with major depressive disorder. J Affect Disord.

[24] Bai S, Xie J, Bai H, Tian T, Zou T, Chen JJ (2021). Gut microbiota-derived inflammation-related serum metabolites as potential biomarkers for major depressive disorder. J Inflamm Res.

[25] Caso JR, MacDowell KS, González-Pinto A, García S, de Diego-Adeliño J, Carceller-Sindreu M (2021). Gut microbiota, innate immune pathways, and inflammatory control mechanisms in patients with major depressive disorder. Transl Psychiatry.

[26] Chahwan B, Kwan S, Isik A, van Hemert S, Burke C, Roberts L (2019). Gut feelings: a randomised, triple-blind, placebo-controlled trial of probiotics for depressive symptoms. J Affect Disord.

[27] Chen YH, Xue F, Yu SF, Li XS, Liu L, Jia YY (2021). Gut microbiota dysbiosis in depressed women: the association of symptom severity and microbiota function. J Affect Disord.

[28] Chen Z, Li J, Gui S, Zhou C, Chen J, Yang C (2018). Comparative metaproteomics analysis shows altered fecal microbiota signatures in patients with major depressive disorder. Neuroreport.

[29] Chung YE, Chen HC, Chou HL, Chen IM, Lee MS, Chuang LC (2019). Exploration of microbiota targets for major depressive disorder and mood related traits. J Psychiatr Res.

[30] Dong Z, Shen X, Hao Y, Li J, Li H, Xu H, et al. Gut microbiome: a potential indicator for differential diagnosis of major depressive disorder and general anxiety disorder. Front Psychiatry 2021;12:651536. 10.3389/fpsyt.2021.651536 PMC8473618 34589003

[31] Fontana A, Manchia M, Panebianco C, Paribello P, Arzedi C, Cossu E, et al. Exploring the role of gut microbiota in major depressive disorder and in treatment resistance to antidepressants. Biomedicines 2020;8:311. 10.3390/biomedicines8090311 PMC7554953 32867257

[32] Huang Y, Shi X, Li Z, Shen Y, Shi X, Wang L (2018). Possible association of Firmicutes in the gut microbiota of patients with major depressive disorder. Neuropsychiatr Dis Treat.

[33] Jiang H, Ling Z, Zhang Y, Mao H, Ma Z, Yin Y (2015). Altered fecal microbiota composition in patients with major depressive disorder. Brain Behav Immun.

[34] Lai WT, Deng WF, Xu SX, Zhao J, Xu D, Liu YH (2021). Shotgun metagenomics reveals both taxonomic and tryptophan pathway differences of gut microbiota in major depressive disorder patients. Psychol Med.

[35] Lin P, Ding B, Feng C, Yin S, Zhang T, Qi X (2017). Prevotella and Klebsiella proportions in fecal microbial communities are potential characteristic parameters for patients with major depressive disorder. J Affect Disord.

[36] Liu P, Gao M, Liu Z, Zhang Y, Tu H, Lei L, et al. Gut microbiome composition linked to inflammatory factors and cognitive functions in first-episode, drug-naive major depressive disorder patients. Front Neurosci 2022;15:800764. 10.3389/fnins.2021.800764 PMC8831735 35153660

[37] Liu RT, Rowan-Nash AD, Sheehan AE, Walsh RFL, Sanzari CM, Korry BJ (2020). Reductions in anti-inflammatory gut bacteria are associated with depression in a sample of young adults. Brain Behav Immun.

[38] Liu Y, Zhang L, Wang X, Wang Z, Zhang J, Jiang R (2016). Similar fecal microbiota signatures in patients with diarrhea-predominant irritable bowel syndrome and patients with depression. Clin Gastroenterol Hepatol.

[39] Mason BL, Li Q, Minhajuddin A, Czysz AH, Coughlin LA, Hussain SK (2020). Reduced anti-inflammatory gut microbiota are associated with depression and anhedonia. J Affect Disord.

[40] Naseribafrouei A, Hestad K, Avershina E, Sekelja M, Linløkken A, Wilson R (2014). Correlation between the human fecal microbiota and depression. Neurogastroenterol Motil.

[41] Rong H, Xie XH, Zhao J, Lai WT, Wang MB, Xu D (2019). Similarly in depression, nuances of gut microbiota: Evidences from a shotgun metagenomics sequencing study on major depressive disorder versus bipolar disorder with current major depressive episode patients. J Psychiatr Res.

[42] Shen Y, Yang X, Li G, Gao J, Liang Y (2021). The change of gut microbiota in MDD patients under SSRIs treatment. Sci Rep.

[43] Stevens BR, Pepine CJ, Richards EM, Kim S, Raizada MK (2021). Depressive hypertension: a proposed human endotype of brain/gut microbiome dysbiosis. Am Heart J.

[44] Stevens BR, Roesch L, Thiago P, Russell JT, Pepine CJ, Holbert RC (2021). Depression phenotype identified by using single nucleotide exact amplicon sequence variants of the human gut microbiome. Mol Psychiatry.

[45] Thapa S, Sheu JC, Venkatachalam A, Runge JK, Luna RA, Calarge CA (2021). Gut microbiome in adolescent depression. J Affect Disord.

[46] Valles-Colomer M, Falony G, Darzi Y, Tigchelaar EF, Wang J, Tito RY (2019). The neuroactive potential of the human gut microbiota in quality of life and depression. Nat Microbiol.

[47] Yang J, Zheng P, Li Y, Wu J, Tan X, Zhou J (2020). Landscapes of bacterial and metabolic signatures and their interaction in major depressive disorders. Sci Adv.

[48] Ye X, Wang D, Zhu H, Wang D, Li J, Tang Y (2021). Gut microbiota changes in patients with major depressive disorder treated with vortioxetine. Front Psychiatry.

[49] Yuan X, Chen B, Duan Z, Xia Z, Ding Y, Chen T (2021). Depression and anxiety in patients with active ulcerative colitis: crosstalk of gut microbiota, metabolomics and proteomics. Gut Microbes.

[50] Zhang Q, Yun Y, An H, Zhao W, Ma T, Wang Z (2021). Gut microbiome composition associated with major depressive disorder and sleep quality. Front Psychiatry.

[51] Zhao H, Jin K, Jiang C, Pan F, Wu J, Luan H (2022). A pilot exploration of multi-omics research of gut microbiome in major depressive disorders. Transl Psychiatry.

[52] Zheng P, Yang J, Li Y, Wu J, Liang W, Yin B (2020). Gut microbial signatures can discriminate unipolar from bipolar depression. Adv Sci.

[53] Zheng S, Zhu Y, Wu W, Zhang Q, Wang Y, Wang Z (2021). A correlation study of intestinal microflora and first-episode depression in Chinese patients and healthy volunteers. Brain Behav.

[54] Zhou Y, Chen C, Yu H, Yang Z (2020). Fecal microbiota changes in patients with postpartum depressive disorder. Front Cell Infect Microbiol.

[55] Sanada K, Nakajima S, Kurokawa S, Barceló-Soler A, Ikuse D, Hirata A (2020). Gut microbiota and major depressive disorder: A systematic review and meta-analysis. J Affect Disord.

[56] Nikolova VL, Hall MRB, Hall LJ, Cleare AJ, Stone JM, Young AH (2021). Perturbations in gut microbiota composition in psychiatric disorders: a review and meta-analysis. JAMA Psychiatry.

[57] Page MJ, McKenzie JE, Bossuyt PM, Boutron I, Hoffmann TC, Mulrow CD (2021). The PRISMA 2020 statement: an updated guideline for reporting systematic reviews. Int J Surg.

[58] Simpson CA, Schwartz OS, Simmons JG (2020). The human gut microbiota and depression: widely reviewed, yet poorly understood. J Affect Disord.

[59] Cheung SG, Goldenthal AR, Uhlemann AC, Mann JJ, Miller JM, Sublette ME (2019). Systematic review of gut microbiota and major depression. Front Psychiatry.

[60] Huang TT, Lai JB, Du YL, Xu Y, Ruan LM, Hu SH (2019). Current understanding of gut microbiota in mood disorders: an update of human studies. Front Genet.

[61] Simpson CA, Diaz-Arteche C, Eliby D, Schwartz OS, Simmons JG, Cowan CSM (2021). The gut microbiota in anxiety and depression - a systematic review. Clin Psychol Rev.

[62] Vindegaard N, Speyer H, Nordentoft M, Rasmussen S, Benros ME (2021). Gut microbial changes of patients with psychotic and affective disorders: a systematic review. Schizophr Res.

[63] Barandouzi ZA, Starkweather AR, Henderson WA, Gyamfi A, Cong XS (2020). Altered composition of gut microbiota in depression: a systematic review. Front Psychiatry.

[64] Shade A (2017). Diversity is the question, not the answer. ISME J.

[65] Ait Chait Y, Mottawea W, Tompkins TA, Hammami R (2020). Unravelling the antimicrobial action of antidepressants on gut commensal microbes. Sci Rep.

[66] Socolar JB, Gilroy JJ, Kunin WE, Edwards DP (2016). How should beta-diversity inform biodiversity conservation?. J Trends Ecol Evol.

[67] Wells G, Shea, B, O’Connell, D, Peterson, J, Welch, V, Losos, M, et al. *The Newcastle-Ottawa Scale (NOS) for Assessing The Quality Of Nonrandomised Studies In Meta-analyses*. 2019.

[68] Hartling L, Milne A, Hamm MP, Vandermeer B, Ansari M, Tsertsvadze A (2013). Testing the Newcastle Ottawa Scale showed low reliability between individual reviewers. J Clin Epidemiol.

[69] McDonald D, Hyde E, Debelius JW, Morton JT, Gonzalez A, Ackermann G (2018). American gut: an open platform for citizen science microbiome research. mSystems.

[70] Bahr SM, Weidemann BJ, Castro AN, Walsh JW, deLeon O, Burnett CM (2015). Risperidone-induced weight gain is mediated through shifts in the gut microbiome and suppression of energy expenditure. EBioMedicine.

[71] Davey KJ, Cotter PD, O’Sullivan O, Crispie F, Dinan TG, Cryan JF (2013). Antipsychotics and the gut microbiome: olanzapine-induced metabolic dysfunction is attenuated by antibiotic administration in the rat. Transl Psychiatry.

[72] Morgan AP, Crowley JJ, Nonneman RJ, Quackenbush CR, Miller CN, Ryan AK (2014). The antipsychotic olanzapine interacts with the gut microbiome to cause weight gain in mouse. PLoS ONE.

[73] Flowers SA, Evans SJ, Ward KM, McInnis MG, Ellingrod VL (2017). Interaction between atypical antipsychotics and the gut microbiome in a bipolar disease cohort. Pharmacotherapy.

[74] Wu GD, Chen J, Hoffmann C, Bittinger K, Chen YY, Keilbaugh SA (2011). Linking long-term dietary patterns with gut microbial enterotypes. Science.

[75] Cumpston M, Li T, Page MJ, Chandler J, Welch VA, Higgins JP (2019). Updated guidance for trusted systematic reviews: a new edition of the Cochrane Handbook for Systematic Reviews of Interventions. Cochrane Database Syst Rev.

[76] Siddaway AP, Wood AM, Hedges LV (2019). How to do a systematic review: a best practice guide for conducting and reporting narrative reviews, meta-analyses, and meta-syntheses. Annu Rev Psychol.

[77] Cheung MW, Cheung SF (2016). Random-effects models for meta-analytic structural equation modeling: review, issues, and illustrations. Res Synth Methods.

[78] Chaimani A, Mavridis D, Salanti G (2014). A hands-on practical tutorial on performing meta-analysis with Stata. Evid Based Ment Health.

[79] Nakagawa S, Cuthill IC (2007). Effect size, confidence interval and statistical significance: a practical guide for biologists. Biol Rev Camb Philos Soc.

[80] Bowden J, Tierney JF, Copas AJ, Burdett S (2011). Quantifying, displaying and accounting for heterogeneity in the meta-analysis of RCTs using standard and generalised Q statistics. BMC Med Res Methodol.

[81] Higgins JP, Thompson SG, Deeks JJ, Altman DG (2003). Measuring inconsistency in meta-analyses. BMJ.

[82] Egger M, Davey SG, Schneider M, Minder C (1997). Bias in meta-analysis detected by a simple, graphical test. BMJ.

[83] Lau J, Ioannidis JP, Schmid CH (1997). Quantitative synthesis in systematic reviews. Ann Intern Med.

[84] Yu M, Jia H, Zhou C, Yang Y, Zhao Y, Yang M (2017). Variations in gut microbiota and fecal metabolic phenotype associated with depression by 16S rRNA gene sequencing and LC/MS-based metabolomics. J Pharm Biomed Anal.

[85] Strandwitz P, Kim KH, Terekhova D, Liu JK, Sharma A, Levering J (2019). GABA-modulating bacteria of the human gut microbiota. Nat Microbiol.

[86] Wan X, Wang W, Liu J, Tong T (2014). Estimating the sample mean and standard deviation from the sample size, median, range and/or interquartile range. BMC Med Res Methodol.

[87] Zhou X, Cao Q, Orfila C, Zhao J, Zhang L (2021). Systematic review and meta-analysis on the effects of astaxanthin on human skin ageing. Nutrients.

[88] Chen HM, Chung YE, Chen HC, Liu YW, Chen IM, Lu ML (2022). Exploration of the relationship between gut microbiota and fecal microRNAs in patients with major depressive disorder. Sci Rep.

[89] Dong Z, Shen X, Hao Y, Li J, Xu H, Yin L (2022). Gut microbiome: a potential indicator for predicting treatment outcomes in major depressive disorder. Front Neurosci.

[90] Han K, Ji L, Wang C, Shao Y, Chen C, Liu L (2022). The host genetics affects gut microbiome diversity in Chinese depressed patients. Front Genet.

[91] Kim SY, Park E, Lim WJ, In Kim S, Jeon SW, Chang Y (2022). Association between gut microbiota and depressive symptoms: a cross-sectional population-based study in South Korea. Psychosom Med.

[92] Kovtun AS, Averina OV, Angelova IY, Yunes RA, Zorkina YA, Morozova AY (2022). Alterations of the composition and neurometabolic profile of human gut microbiota in major depressive disorder. Biomedicines.

[93] Li X, Jing K, Lu H, Li K, Zhang Y, Hasichaolu. Exploring the correlation between changes in gut microbial community diversity and depression in human populations. BioMed Research International 2022;2022:6334868. 10.1155/2022/6334868 PMC9355758 35937392

[94] Sun N, Zhang J, Wang J, Liu Z, Wang X, Kang P (2022). Abnormal gut microbiota and bile acids in patients with first‐episode major depressive disorder and correlation analysis. Psychiatry Clin Neurosci.

[95] Tsai CF, Chuang CH, Wang YP, Lin YB, Tu PC, Liu PY (2022). Differences in gut microbiota correlate with symptoms and regional brain volumes in patients with late-life depression. Front Aging Neurosci.

[96] Zhang Y, Fan Q, Hou Y, Zhang X, Yin Z, Cai X (2022). Bacteroides species differentially modulate depression-like behavior via gut-brain metabolic signaling. Brain Behav Immun.

[97] Zhou YY, Zhang X, Pan LY, Zhang WW, Chen F, Hu SS (2022). Fecal microbiota in pediatric depression and its relation to bowel habits. J Psychiatr Res.

[98] Ai D, Pan H, Li X, Gao Y, Liu G, Xia LC (2019). Identifying gut microbiota associated with colorectal cancer using a zero-inflated lognormal model. Front Microbiol.

[99] Gong D, Gong X, Wang L, Yu X, Dong Q (2016). Involvement of reduced microbial diversity in inflammatory bowel disease. Gastroenterol Res Pract.

[100] Ma B, Liang J, Dai M, Wang J, Luo J, Zhang Z (2019). Altered gut microbiota in chinese children with autism spectrum disorders. Front Cell Infect Microbiol.

[101] Nguyen TT, Hathaway H, Kosciolek T, Knight R, Jeste DV (2021). Gut microbiome in serious mental illnesses: A systematic review and critical evaluation. Schizophr Res.

[102] Prehn-Kristensen A, Zimmermann A, Tittmann L, Lieb W, Schreiber S, Baving L (2018). Reduced microbiome alpha diversity in young patients with ADHD. PLoS ONE.

[103] Belizário JE, Faintuch J, Garay-Malpartida M (2018). Gut microbiome dysbiosis and immunometabolism: new frontiers for treatment of metabolic diseases. Mediat Inflamm.

[104] Loubinoux J, Bronowicki JP, Pereira IA, Mougenel JL, Faou AE (2002). Sulfate-reducing bacteria in human feces and their association with inflammatory bowel diseases. FEMS Microbiol Ecol.

[105] Pedersen C, Ijaz UZ, Gallagher E, Horton F, Ellis RJ, Jaiyeola E (2018). Fecal Enterobacteriales enrichment is associated with increased in vivo intestinal permeability in humans. Physiol Rep.

[106] Raison CL, Capuron L, Miller AH (2006). Cytokines sing the blues: inflammation and the pathogenesis of depression. Trends Immunol.

[107] Vogelzangs N, Duivis HE, Beekman AT, Kluft C, Neuteboom J, Hoogendijk W (2012). Association of depressive disorders, depression characteristics and antidepressant medication with inflammation. Transl Psychiatry.

[108] Kiecolt-Glaser JK, Derry HM, Fagundes CP (2015). Inflammation: depression fans the flames and feasts on the heat. Am J Psychiatry.

[109] Li B, Guo K, Zeng L, Zeng B, Huo R, Luo Y (2018). Metabolite identification in fecal microbiota transplantation mouse livers and combined proteomics with chronic unpredictive mild stress mouse livers. Transl Psychiatry.

[110] Louis P, Flint HJ (2017). Formation of propionate and butyrate by the human colonic microbiota. Environ Microbiol.

[111] Sokol H, Pigneur B, Watterlot L, Lakhdari O, Bermúdez-Humarán LG, Gratadoux JJ (2008). Faecalibacterium prausnitzii is an anti-inflammatory commensal bacterium identified by gut microbiota analysis of Crohn disease patients. Proc Natl Acad Sci USA.

[112] Vital M, Karch A, Pieper DH (2017). Colonic butyrate-producing communities in humans: an overview using omics data. mSystems.

[113] Sakon H, Nagai F, Morotomi M, Tanaka R (2008). Sutterella parvirubra sp. nov. and Megamonas funiformis sp. nov., isolated from human faeces. Int J Syst Evol Microbiol.

[114] Katano Y, Fujinami S, Kawakoshi A, Nakazawa H, Oji S, Iino T. et al. Complete genome sequence of Oscillibacter valericigenes Sjm18-20T (=NBRC 101213T)[J.Stand Genomic Sci.2012;6:406–414. 10.4056/sigs.2826118 PMC3558957 23408234

[115] Ben-Ari Y (2013). Neuropaediatric and neuroarchaeology: understanding development to correct brain disorders. Acta Paediatr.

[116] David LA, Maurice CF, Carmody RN, Gootenberg DB, Button JE, Wolfe BE (2014). Diet rapidly and reproducibly alters the human gut microbiome. Nature.

[117] Rinninella E, Raoul P, Cintoni M, Franceschi F, Miggiano GAD, Gasbarrini A (2019). What is the healthy gut microbiota composition? A changing ecosystem across age, environment, diet, and diseases. Microorganisms.

[118] Rea K, Dinan TG, Cryan JF (2016). The microbiome: a key regulator of stress and neuroinflammation. Neurobiol Stress.

[119] Yatsunenko T, Rey FE, Manary MJ, Trehan I, Dominguez-Bello MG, Contreras M (2012). Human gut microbiome viewed across age and geography. Nature.

[120] Lim ES, Zhou Y, Zhao G, Bauer IK, Droit L, Ndao IM (2015). Early life dynamics of the human gut virome and bacterial microbiome in infants. Nat Med.

[121] Vemuri R, Gundamaraju R, Shastri MD, Shukla SD, Kalpurath K, Ball M (2018). Gut microbial changes, interactions, and their implications on human lifecycle: an ageing perspective. Biomed Res Int.

[122] Claesson MJ, Cusack S, O’Sullivan O, Greene-Diniz R, de Weerd H, Flannery E (2011). Composition, variability, and temporal stability of the intestinal microbiota of the elderly. Proc Natl Acad Sci USA.

[123] Chen JJ, He S, Fang L, Wang B, Bai SJ, Xie J (2020). Age-specific differential changes on gut microbiota composition in patients with major depressive disorder. Aging.

[124] Ma ZS, Li W (2019). How and why men and women differ in their microbiomes: medical ecology and network analyses of the microgenderome. Adv Sci.

[125] Chen JJ, Zheng P, Liu YY, Zhong XG, Wang HY, Guo YJ (2018). Sex differences in gut microbiota in patients with major depressive disorder. Neuropsychiatr Dis Treat.

[126] Hawinkel S, Mattiello F, Bijnens L, Thas O (2019). A broken promise: microbiome differential abundance methods do not control the false discovery rate. Brief Bioinform.

