# Supplementary material for: Gut microbiota composition in depressive disorder: a systematic review, meta-analysis, and meta-regression
Source: Transl Psychiatry. 2023 Dec 8;13:379. doi: 10.1038/s41398-023-02670-5 (PMC10709466; doi:10.1038/s41398-023-02670-5)
Supplement: Supplementary file 1 — Supplementary materials [file 41398_2023_2670_MOESM1_ESM.docx]

**Supplemental materials**

Table S1. Quality Assessment of Included Studies.

Table S2. Stool sample collection, storage, and DNA extraction procedures of the included studies.

Table S3. Methodology and findings of the included studies assessing alpha diversity.

Table S4. Between-group meta-regressions, by moderator.

Table S5. Methodology and findings of the included studies assessing beta diversity.

Figure S1. Meta-regression of Hamilton Depression Rating Scale scores on Chao1 index, Shannon index, Firmicutes and Bacteroidetes.

Figure S2. Changes in Relative Abundance of Genus Level Reported by at Least 2 Studies

Figure S3. Sensitivity analyses for the alpha diversity, the phylum Firmicutes and Bacteroidetes meta-analyses.

Figure S4. Publication bias assessment for the alpha diversity, the phylum Firmicutes and Bacteroidetes meta-analyses.

**Table S1. Quality Assessment of Included Studies**

| First Author (Year) | Selection | Comparability | Exposure | Total |
| --- | --- | --- | --- | --- |
| Aizawa et al (2016)^23^ | 1a | 1a，1b | 1a | 4 |
| Bai et al (2021)^24^ | 1a，4a | 1a | 1a，2a | 5 |
| Caso et al (2021)^25^ | 1a，4a | 1a，1b | 2a | 5 |
| Chahwan et al (2019)^26^ | 1a，3a，4a | 1a，1b | 1a，2a | 7 |
| Chen et al (2018)^28^ | 1a，4a | 1a，1b | 2a | 5 |
| Chen et al (2021)^27^ | 1a，4a | 1a，1b |  | 4 |
| Chen et al (2022)^88^ | 1a，3a，4a | 1a，1b | 2a | 6 |
| Chung et al (2019)^29^ | 1a，3a，4a | 1a，1b | 2a | 6 |
| Dong et al (2021)^30^ | 1a，3a，4a | 1a，1b |  | 5 |
| Dong et al (2022)^89^ | 1a，2a，4a | 1a，1b | 2a | 6 |
| Fontana et al (2020)^31^ | 1a，3a，4a | 1a，1b |  | 5 |
| Han et al (2022)^90^ | 1a，2a，4a | 1a，1b | 2a | 6 |
| Huang et al (2018)^32^ | 1a，2a，4a | 1a，1b |  | 5 |
| Jiang et al (2015)^33^ | 1a，2a，4a | 1a，1b |  | 5 |
| Kelly et al (2016)^22^ | 1a，3a，4a | 1a，1b | 2a | 6 |
| Kim et al (2022)^91^ | 1a，2a，4a | 1a | 2a | 5 |
| Kovtun et al (2022)^92^ | 1a，4a | 1a，1b | 2a | 5 |
| Lai et al (2021)^34^ | 1a，3a，4a | 1a，1b | 1b | 6 |
| Li et al (2022a)^93^ | 1a，2a，4a | 1a，1b |  | 5 |
| Lin et al (2017)^35^ | 1a，4a | 1a |  | 3 |
| Liu et al (2016)^38^ | 2a，3a，4a | 1a，1b | 2a | 6 |
| Liu et al (2020)^37^ | 1a，2a，3a，4a | 1a，1b | 1b，2a | 8 |
| Liu et al (2022)^36^ | 1a，4a | 1a，1b | 2a | 5 |
| Mason et al (2020)^39^ | 1a，3a，4a | 1a，1b | 1b，2a | 7 |
| Naseribafrouei et al (2014)^40^ | 1a，4a | 1a，1b | 2a | 5 |
| Rong et al (2019)^41^ | 1a，3a，4a | 1a，1b |  | 5 |
| Shen et al (2021)^42^ | 1a，3a，4a | 1a，1b | 1b，2a | 7 |
| Stevens et al (2021a)^44^ | 1a，4a | 1a，1b |  | 4 |
| Stevens et al (2021b)^43^ | 1a，4a | 1a，1b |  | 4 |
| Sun et al (2022)^94^ | 2a，4a | 1a，1b | 2a | 5 |
| Thapa et al (2021)^45^ | 1a，3a，4a | 1a，1b |  | 5 |
| Tsai et al (2022)^95^ | 1a，3a，4a | 1a，1b | 2a | 6 |
| Valles-Colomer et al (2019)^46^ | 4a | 1a，1b |  | 3 |
| Yang et al (2020)^47^ | 1a，2a，3a，4a | 1a，1b | 1b，2a | 8 |
| Ye et al (2021)^48^ | 1a，3a，4a | 1a，1b | 1b | 6 |
| Yuan et al (2021)^49^ | 1a，2a，4a | 1a，1b | 2a | 6 |
| Zhang et al (2021)^50^ | 4a | 1a | 2a | 3 |
| Zhang et al (2022)^96^ | 4a | 1a，1b |  | 3 |
| Zhao et al (2022)^51^ | 1a，2a，3a，4a | 1a，1b | 2a | 7 |
| Zheng et al (2016)^19^ | 1a，2a，3a，4a | 1a，1b | 1b，2a | 8 |
| Zheng et al (2020)^52^ | 1a，4a | 1a，1b | 2a | 5 |
| Zheng et al (2021)^53^ | 1a，3a，4a | 1a，1b |  | 5 |
| Zhou et al (2020)^54^ | 2a，4a | 1a，1b | 2a | 5 |
| Zhou et al (2022)^97^ | 1a，2a，3a，4a | 1a，1b | 2a | 7 |

**Table S2. Stool sample collection, storage and DNA extraction procedures of the included studies**

| Study | Collection & handling by participant | Long-term storage | DNA extraction method |
| --- | --- | --- | --- |
| Aizawa et al (2016)^23^ | Collected with RNA stabilizer and stored at room temperature or at 4°C until sent to lab | at 4°C until use | Total RNA fractions were extracted (Yakult Central Institute), method not reported |
| Bai et al  (2021)^24^ | Nr | Nr | Standard protocols by Majorbio Bio-Pharm Technology Co. Ltd. (Shanghai, China) |
| Caso et al  (2021)^25^ | Sterile plastic cups were used to collect samples and stored in an icebox | at −80 °C until use | QIAamp© DNA Stool Mini Kit (Qiagen, Hilden, Germany) according to the manufacturer’s instructions |
| Chahwan et al (2019)^26^ | Kept on ice or refrigerated before delivery to study staff | samples placed at 4 °C and aliquots stored at −80 °C several days | PowerFecal DNA Isolation Kit (MoBio), following manufacturer's instructions |
| Chen et al  (2018)^28^ | Sterile plastic cups were used to collect samples | at −80 °C until use | Tandem mass spectra data were extracted and analyzed using Mascot (Matrix Science) against a combined Swiss prot-human-20151226 and TrEMBL bacteria database |
| Chen et al  (2021)^27^ | Sterile plastic cups were used to collect samples and stored at −80 °C within 0.5 hour | at −20 °C until use | Qiagen QIAamp DNA Stool Mini Kit (Qiagen) according to manufacturer’s instructions |
| Chen et al(2022)^88^ | Kept on refrigerated before delivery to study staff | at −80 °C until use | PowerMicrobiome™ RNA Isolation Kit (MoBio, Germany) |
| Chung et al  (2019)^29^ | Delivered in 4 °C | at −80 °C until use | QIAamp DNA Stool Mini Kit (QIAGEN Inc.,USA) or phenolchloroform extraction method |
| Dong et al  (2021)^30^ | Sterile plastic cup was used to collect sample and immediately frozen. | at −80 °C until use | QIAamp DNA stool mini kit (Qiagen, Hilden, Germany) according to manufacturer instructions |
| Dong et al (2022)^89^ | Nr | at −80 °C until use | QIAamp DNA Stool Mini Kit (Qiagen, Hilden, Germany) |
| Fontana et al (2020)^31^ | Collected with DNA stabilization buffer (Canvax Biotech, Cordoba, Spain) | at −80 °C until use | QIAamp DNA Stool Mini Kit (Qiagen, Milan, Italy) according to the manufacturer’s protocol |
| Han et al  (2022)^90^ | Sterile plastic cup was used to collect samples | at −80 °C until use | QIAamp DNA Stool Mini Kit (Qiagen, Hilden,Germany) according to the manufacturer’s instructions |
| Huang et al  (2018)^32^ | Sterile containers were used to collect samples | at −80 °C until use | PowerSoil DNA Kit (Missouri Biotechnology Association,Jefferson, MO, USA) according to the manufacturer’s instructions. |
| Jiang et al  (2015)^33^ | Sterile plastic cups were used to collect samples and kept in an icebox | at −80 °C until use | QIAamp® DNA Stool Mini Kit (Qiagen, Hilden, Germany), following manufacturer's instructions, with additional glass-bead beating steps on a Mini-beadbeater (FastPrep; Thermo Electron Corp., Boston,MA,USA) |
| Kelly et al  (2016)^22^ | Collected in plastic containers containing an anaerobic generator AnaeroGen Compact Oxoid sachet | homogenized, aliquoted and stored at −80 °C until further use | QIAamp DNA Stool Mini Kit (QIAGEN) |
| Kim et al  (2022)^91^ | Sterile, dry, screw cap containers were used to collect samples and immediately frozen at −20°C | at −70 °C until use | MOBio PowerSoil DNA Isolation Kit (MOBIO Laboratories, Carlsbad, California) according to the manufacturer’s instructions |
| Kovtun et al (2022)^92^ | Nr | at −80 °C until use | QIAamp PowerFecal Pro DNA Kit (Qiagen, Hilden, Germany) according to the manufacturer’s instructions |
| Lai et al (2021)^34^ | Nr | immediately stored at −80 °C until use | StoolGen DNA kit (CWBiotech Co.,Beijing, China) |
| Li et al (2022)^93^ | Sterile plastic box was used to collect samples | at −80 °C until use | StoolGen DNA kit (Beijing Youji Technology Co., Ltd.) |
| Lin et al  (2017)^35^ | Nr | immediately stored at −70 °C until use | Tiagen DNA Stool Mini Kit (Tiagen Biotech,Beijing,China), following manufacturer's protocols |
| Liu et al (2016)^38^ | Nr | immediately stored at −80 °C until use | PowerSoil DNA Isolation Kit (MoBio Laboratories, Inc. CA) |
| Liu et al  (2020)^37^ | OMNIgene•GUT stool collection kits (DNA Genotek) | immediately aliquoted and stored at −80°C until use | ZymoBIOMICS 96 DNA Kit (Zymo Research) according to manufacturer’s protocols |
| Liu et al  (2022)^36^ | Nr | immediately stored at −80 °C until use | QIAamp® DNA Stool Mini Kit (Qiagen, Hilden, Germany), following maker's instructions |
| Mason et al (2020)^39^ | Frozen in-home freezer after collection | at −80 °C until use | RNAseA (QIAGEN) and column-purified (PCR Purification Kit, QIAGEN) |
| Naseribafrouei et al (2014)^40^ | Outpatient samples frozen at −20 °C in home freezer and transported at below zero to centralized - 70 °C storage.  Inpatients stored directly at -70 °C. | at −80 °C until use | MagTM mini kit (LGC, Middlesex, UK) following the manufacturer’s recommendations |
| Rong et al (2019)^41^ | Nr | at −80 °C until use | StoolGen DNA kit (CWBiotech Co.,Beijing, China) |
| Shen et al (2021)^42^ | A clean container was used to collect samples | at −80 °C until use | PowerSoil DNA kit (MoBio, USA) according to manufacturer’s instructions |
| Stevens et al (2021a)^44^ | Collected with OMNIgeneGUT fecal collection kits (DNAGenotek, Ottawa, Ontario, Canada) | at −80 °C until use | E.Z.N.A Stool Extraction Kit following the manufacturer’s protocol (Omega Bio-tek, Doraville, CA). |
| Stevens et al (2021b)^43^ | collected with OMNIgene GUT fecal collection kits (DNAGenotek, Ottawa, Ontario, Canada) | at −80 °C until use | PowerFecal DNA extraction kit (MoBio) following manufacturer’s instructions |
| Sun et al (2022)^94^ | Stool collection tubes were used to collect samples | at −80 °C until use | Nr |
| Thapa et al  (2021)^45^ | Aliquoted and frozen within 15-30 min in the lab.  Placed on dry ice and picked up within 24 h if the sample was provided at home. | at −80 °C until use | MoBio PowerSoil kit (Qiagen, Hilden, Germany) following manufacturer recommendations |
| Tsai et al (2022)^95^ | Fresh stool specimens were self-collected | at −80 °C until use | Nr |
| Valles-Colomer et al (2019)^46^ | Nr | Nr | PowerMicrobiome® RNA Isolation Kit (MoBio Laboratories Inc., Carlsbad, CA) following manufacturer’s instruction |
| Yang et al  (2020)^47^ | Nr | Nr | E.Z.N.A. Soil DNA Kit (Omega Bio-tek, Norcross, GA, USA) according to manufacturer’s instructions |
| Ye et al  (2021)^48^ | Collected with disposable feces collectors and frozen in an ultra-low temperature freezer at−80 °C | at −80 °C until use | Power Soil DNA Isolation Kit (MO BIO Laboratories, Carlsbad, CA, USA) following the manufacturer’s protocol |
| Yuan et al  (2021)^49^ | Stool sample collection kit (Beijing Allwegene Technology Co.,Ltd.,China) | at -80℃ within two hours,until use | E.Z.N.A.R Stool DNA Kit (Omega Bio-tek, Norcross, GA, U.S.A.) |
| Zhang et al  (2021)^50^ | Sample tube containing RNA stabilization solution (TinyGen, Bio-Tech,Shanghai, China) | at −80 °C until use | Nr |
| Zhang et al  (2022)^96^ | Fecal samples collected and placed in RNA stabilization solution (TinyGen, Bio-Tech, Shanghai, China) | at −80 °C until use | DNA gel extraction kit (Axygen, USA) |
| Zhao et al (2022)^51^ | Nr | at −80 °C until use | Standard Stool Extraction Kit protocol |
| Zheng et al  (2016)^19^ | Nr | immediately stored at −80 °C until use | Standard Power Soil Kit protocol |
| Zheng et al  (2020)^52^ | Nr | Nr | OMEGA-soil DNA Kit (Omega Bio-Tek, USA) according to manufacturer’s protocols |
| Zheng et al  (2021)^53^ | Stool collection tube with preservation solution | at −80 °C until use | QIAamp DNA Stool Mini Kit (QIAGEN) following the manufacturer's instructions |
| Zhou et al  (2020)^54^ | Collected with sterile plastic cup and immediately stored at −20°C | at −80 °C until use | MOBIO PowerSoil® DNA Isolation kit |
| Zhou et al  (2022)^97^ | Nr | immediately stored at −80 °C until use | FastDNA SPIN Kit for Feces (MP Biomedicals, Santa Ana, CA, USA) according to the manufacturer’s instructions |

**Table S3. Methodology and findings of the included studies assessing Alpha diversity**

| Study | Analysis | Finding |
| --- | --- | --- |
| Bai et al (2021)^24^ | Chao1., Shannon, Simpson，PD | no sig. difference |
| Caso et al (2021)^25^ | Shannon | no sig. difference |
| Chahwan et al (2019)^26^ | Observed sp., Chao1, Shannon | no sig. difference |
| Chen et al (2021)^27^ | Observed sp., Chao1, ACE, Shannon, Simpson | no sig. difference |
| Chen et al (2022)^88^ | Richness, Shannon | no sig. difference |
| Chung et al (2019)^29^ | Observed sp., Chao1, Shannon, PD | no sig. difference |
| Dong et al (2021)^30^ | ACE, Chao1,Simpson，Shannon | no sig. difference |
| Dong et al (2022)^89^ | Chao1, Shannon | no sig. difference |
| Han et al (2022)^90^ | Chao1, ACE  Shannon, Simpson | lower  no sig. difference |
| Huang et al (2018)^32^ | Chao1, ACE, Shannon, PD | lower |
| Jiang et al (2015)^33^ | Shannon  Chao1, ACE, Simpson，Evenness | higher  no sig. difference |
| Kelly et al (2016)^22^ | Observed sp., Chao1,PD  Shannon | lower no sig. difference |
| Kim et al (2022)^91^ | Observed ASVs., Pielou’s evenness, Shannon, PD | no sig. difference |
| Kovtun et al (2022)^92^ | Shannon | no sig. difference |
| Lai et al (2019)^34^ | Fisher  Shannon | lower  no sig. difference |
| Li et al (2022)^93^ | Chao1, ACE,Shannon, Simpson | lower |
| Liu et al (2016)^38^ | Shannon | lower |
| Liu et al (2020)^37^ | Observed sp., Shannon,  PD | no sig. difference  lower |
| Liu et al (2022)^36^ | Observed sp., Chao1,Shannon, PD  Simpson, Pielou’s evenness | no sig. difference  lower |
| Naseribafrouei et al (2014)^40^ | Observed sp.  Simpsons | higher  no sig. difference |
| Rong et al (2019)^41^ | Chao 1,Gm coefficient  Shannon, Inv. Simpson | lower  no sig. difference |
| Shen et al (2021)^42^ | Ace, Chao1, Shannon  Simpson | higher  lower |
| Sun et al (2022)^94^ | Chao1,Shannon, PD, Observed sp.  Simpson, Pielou’s evenness | no sig. difference  lower |
| Thapa et al (2021)^45^ | Observed sp., Chao1, ACE, Shannon, PD | no sig. difference |
| Tsai et al (2022)^95^ | Shannon, PD | lower |
| Yang et al (2020)^47^ | Chao1, Shannon, Inv. Simpson | no sig. difference |
| Ye et al (2021)^48^ | Chao1, Shannon | higher |
| Yuan et al (2021)^49^ | Shannon, PD | lower |
| Zhang et al (2021)^50^ | Chao 1, ACE, Shannon, Simpson | no sig. difference |
| Zhang et al (2022)^96^ | Chao1, ACE, Observed sp. | lower |
| Zheng et al (2016)^19^ | Observed sp., Shannon, Simpson, PD | no sig. difference |
| Zheng et al (2020)^52^ | Chao1, ACE, Shannon, Inv. Simpson | no sig. difference |
| Zheng et al (2021)^53^ | Chao1, ACE, Shannon, Simpson, Sobe | no sig. difference |
| Zhou et al (2020)^54^ | Observed sp., Shannon, PD, Evenness | no sig. difference |
| Zhou et al (2022)^97^ | Chao1, ACE, Shannon, Sobs | lower |

**Table S4. Between-group meta-regressions, by moderator**

|  | Number of studies | N | | Meta-regression | | | |
| --- | --- | --- | --- | --- | --- | --- | --- |
|  |  | P | HC | Coefficient | SE | 95%CI | P value |
| Chao1 |  |  |  |  |  |  |  |
| Regional variations(east/west) | 19 | 1045 | 930 | 1.70 | 3.96 | -6.66 to 10.05 | 0.67 |
| Use of psychotropic medication | 19 | 1045 | 930 | -2.05 | 2.48 | -7.29 to 3.19 | 0.42 |
| Age | 19 | 1045 | 930 | 0.11 | 0.13 | -0.17 to 0.38 | 0.43 |
| Sex (proportion of women) | 19 | 1045 | 930 | -0.02 | 0.09 | -0.20 to 0.16 | 0.80 |
| BMI | 16 | 855 | 851 | 0.53 | 0.92 | -1.44 to 2.50 | 0.57 |
| HDRS scores | 15 | 817 | 824 | 0.18 | 0.42 | -0.72 to 1.08 | 0.68 |
| Shannon |  |  |  |  |  |  |  |
| Regional variations(east/west) | 29 | 1506 | 2293 | 0.15 | 0.68 | -1.25 to 1.56 | 0.82 |
| Use of psychotropic medication | 29 | 1506 | 2293 | -0.30 | 0.52 | -1.36 to 0.76 | 0.57 |
| Age | 29 | 1506 | 2293 | 0.002 | 0.02 | -0.05 to 0.05 | 0.94 |
| Sex (proportion of women) | 29 | 1506 | 2293 | -0.009 | 0.02 | -0.04 to 0.03 | 0.62 |
| BMI | 25 | 1267 | 2152 | 0.07 | 0.22 | -0.39 to 0.54 | 0.74 |
| HDRS scores | 21 | 1017 | 1012 | 0.08 | 0.07 | -0.07 to 0.24 | 0.28 |
| Firmicutes |  |  |  |  |  |  |  |
| Regional variations(east/west) | 18 | 665 | 609 | 0.54 | 1.03 | -1.64 to 2.71 | 0.61 |
| Use of psychotropic medication | 18 | 665 | 609 | 1.82 | 0.83 | 0.05 to 3.59 | 0.05 |
| Age | 18 | 665 | 609 | 0.02 | 0.04 | -0.08 to 0.11 | 0.73 |
| Sex (proportion of women) | 18 | 665 | 609 | -0.03 | 0.03 | -0.09 to 0.03 | 0.31 |
| BMI | 14 | 440 | 460 | -0.02 | 0.09 | -0.22 to 0.19 | 0.87 |
| HDRS scores | 12 | 400 | 431 | 0.05 | 0.07 | -0.12 to 0.21 | 0.54 |
| Bacteroidetes |  |  |  |  |  |  |  |
| Regional variations(east/west) | 18 | 760 | 753 | -0.74 | 1.20 | -3.28 to 1.80 | 0.54 |
| Use of psychotropic medication | 18 | 760 | 753 | -1.68 | 1.08 | -3.96 to 0.60 | 0.14 |
| Age | 18 | 760 | 753 | -0.01 | 0.05 | -0.13 to 0.10 | 0.78 |
| Sex (proportion of women) | 18 | 760 | 753 | 0.03 | 0.03 | -0.04 to 0.10 | 0.40 |
| BMI | 14 | 535 | 604 | -0.20 | 0.47 | -1.22 to 0.82 | 0.68 |
| HDRS scores | 13 | 522 | 602 | -0.02 | 0.10 | -0.22 to 0.19 | 0.85 |

**Table S5. Methodology and findings of the included studies assessing beta diversity**

| Study | Metric | Analysis | Finding |
| --- | --- | --- | --- |
| Bai et al (2021)^24^ | Nr | PCoA | sig. different |
| Caso et al (2021)^25^ | Jaccard Bray-Curtis | PCoA, PERMANOVA | no sig. difference no sig. difference |
| Chahwan et al (2019)^26^ | Weighted Unifrac | PCoA, PERMANOVA | no sig. difference |
| Chen et al (2021)^27^ | Unweighted Unifrac  Weighted Unifrac | PCoA | sig. different sig. different |
| Chen et al (2022)^88^ | Bray-Curtis | PCoA | no sig. difference |
| Chung et al (2019)^29^ | Unweighted Unifrac  Weighted Unifrac | PERMANOVA | sig. different |
| Dong et al (2021)^30^ | Bray-Curtis | PCA Hierarchical clustering tree | no sig. difference |
| Dong et al (2022)^89^ | Bray-Curtis | PCoA, ANOSIM | no sig. difference |
| Han et al (2022)^90^ | Bray-Curtis | PCoA , NMDS | no sig. difference |
| Huang et al (2018)^32^ | Unweighted Unifrac  Weighted Unifrac | PCoA | no sig. difference sig. different |
| Jiang et al (2015)^33^ | Unweighted Unifrac | PCoA | no sig. difference |
| Kelly et al (2016)^22^ | Bray-Curtis  Unweighted Unifrac  Weighted Unifrac | PCoA, Adonis  multivariate ANOVA | sig. different  sig. different  sig. different |
| Kim et al (2022)^91^ | Bray-Curtis  Jaccard  Unweighted Unifrac  Weighted Unifrac | PCoA, PERMANOVA | sig. Different  no sig. difference no sig. difference no sig. difference |
| Kovtun et al (2022)^92^ | Nr | NMDS，PERMANOVA | sig. different |
| Lai et al (2021)^34^ | Bray-Curtis | PCoA, PERMANOVA | sig. different |
| Lin et al (2017)^35^ | Weighted Unifrac | PCoA | sig. different |
| Liu et al (2020)^37^ | Unweighted Unifrac  Weighted Unifrac Bray - Curtis | PCoA | sig. different  sig. different  sig. different |
| Liu et al (2022)^36^ | Jaccard | PCoA | sig. different |
| Mason et al (2020)^39^ | Weighted Unifrac | PERMANOVA | no sig. difference |
| Rong et al (2019)^41^ | Bray-Curtis | PCoA | no sig. difference |
| Shen et al (2021)^42^ | Jaccard | PCoA | no sig. difference |
| Stevens et al (2021a)^44^ | Bray-Curtis | PCoA, PERMANOVA | no sig. difference |
| Sun et al (2022)^94^ | Nr | NMDS, ANOSIM | sig. different |
| Thapa et al (2021)^45^ | Bray-Curtis  Unweighted Unifrac  Weighted Unifrac Aitchison | PCoA, PERMANOVA PCoA, PERMANOVA PCoA, PERMANOVA PCA, PERMANOVA | no sig. difference no sig. difference no sig. difference no sig. difference |
| Tsai et al (2022)^95^ | UniFrac (unweighted) | PCoA | sig. different |
| Yang et al (2020)^47^ | Bray-Curtis | PCoA, PERMANOVA | sig. different |
| Ye et al (2021)^48^ | unweighted UniFrac | PCoA | sig. different |
| Zhang et al (2021)^50^ | Bray-Curtis  Unweighted Unifrac  Weighted Unifrac Jaccard | PCoA, ANOSIM | no sig. difference no sig. difference sig. different sig. different |
| Zhang et al (2022)^96^ | Bray-Curtis | PCoA, PERMANOVA | sig. different |
| Zhao et al (2022)^51^ | Nr | PCA | sig. different |
| Zheng et al (2016)^19^ | Unweighted Unifrac  Weighted Unifrac | PCoA | sig. different sig. different |
| Zheng et al (2020)^52^ | PLS-DA | PERMANOVA | sig. different |
| Zhou et al (2020)^54^ | Weighted Unifrac | PCoA | sig. different |
| Zhou et al (2022)^97^ | Weighted Unifrac | PCoA | sig. different |

PCoA = principal coordinates analysis; PERMANOVA = permutational analysis of variance; PLS-DA = principal least squares

discriminant analysis; OPLS-DA = orthogonal principal least squares discriminant analysis; PCA= principal component analysis;

ANOSIM= analysis of similarities

**
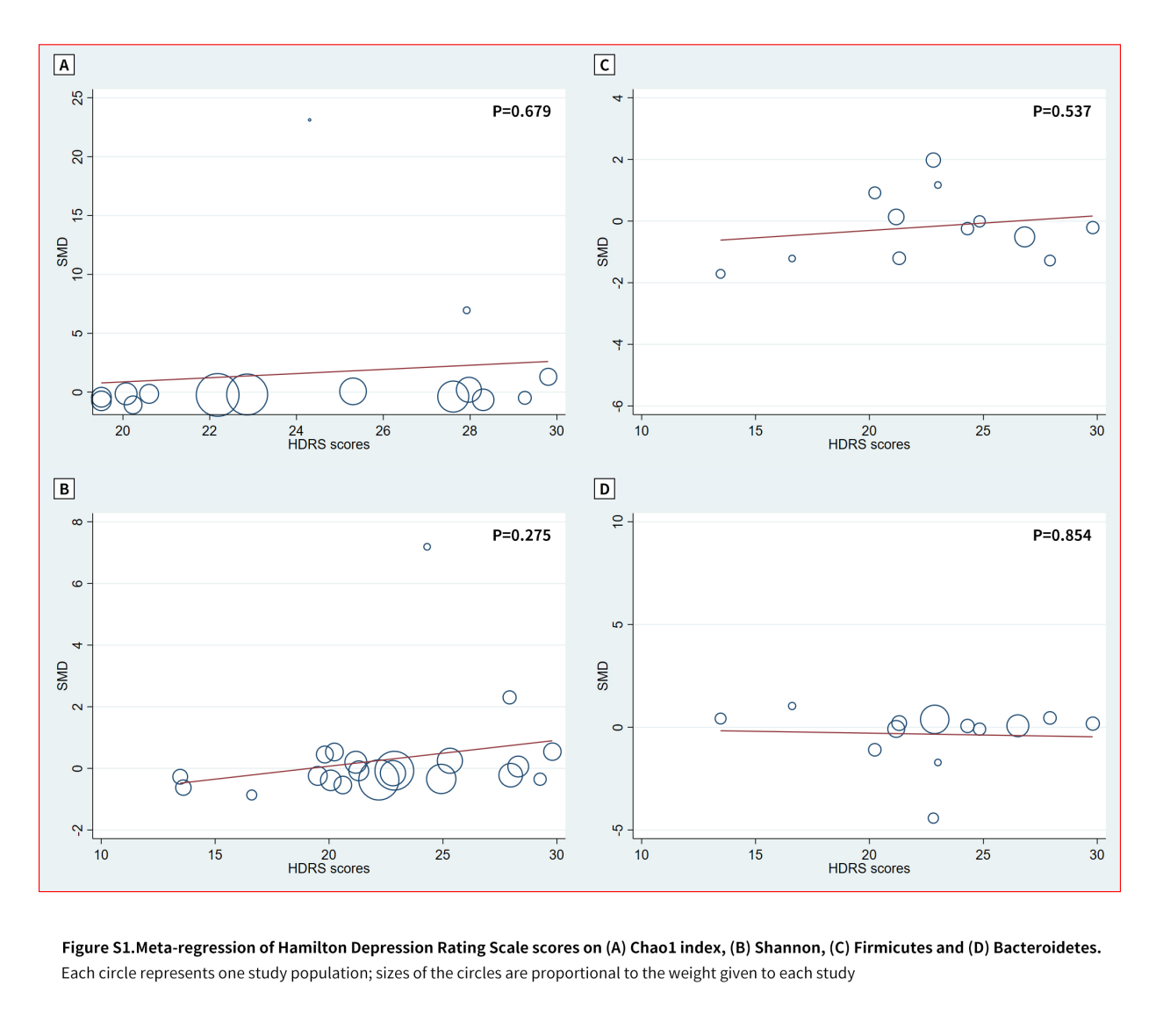
**

**Figure S1. Meta-regression of Hamilton Depression Rating Scale scores on Chao1 index, Shannon index, Firmicutes and Bacteroidetes**

**
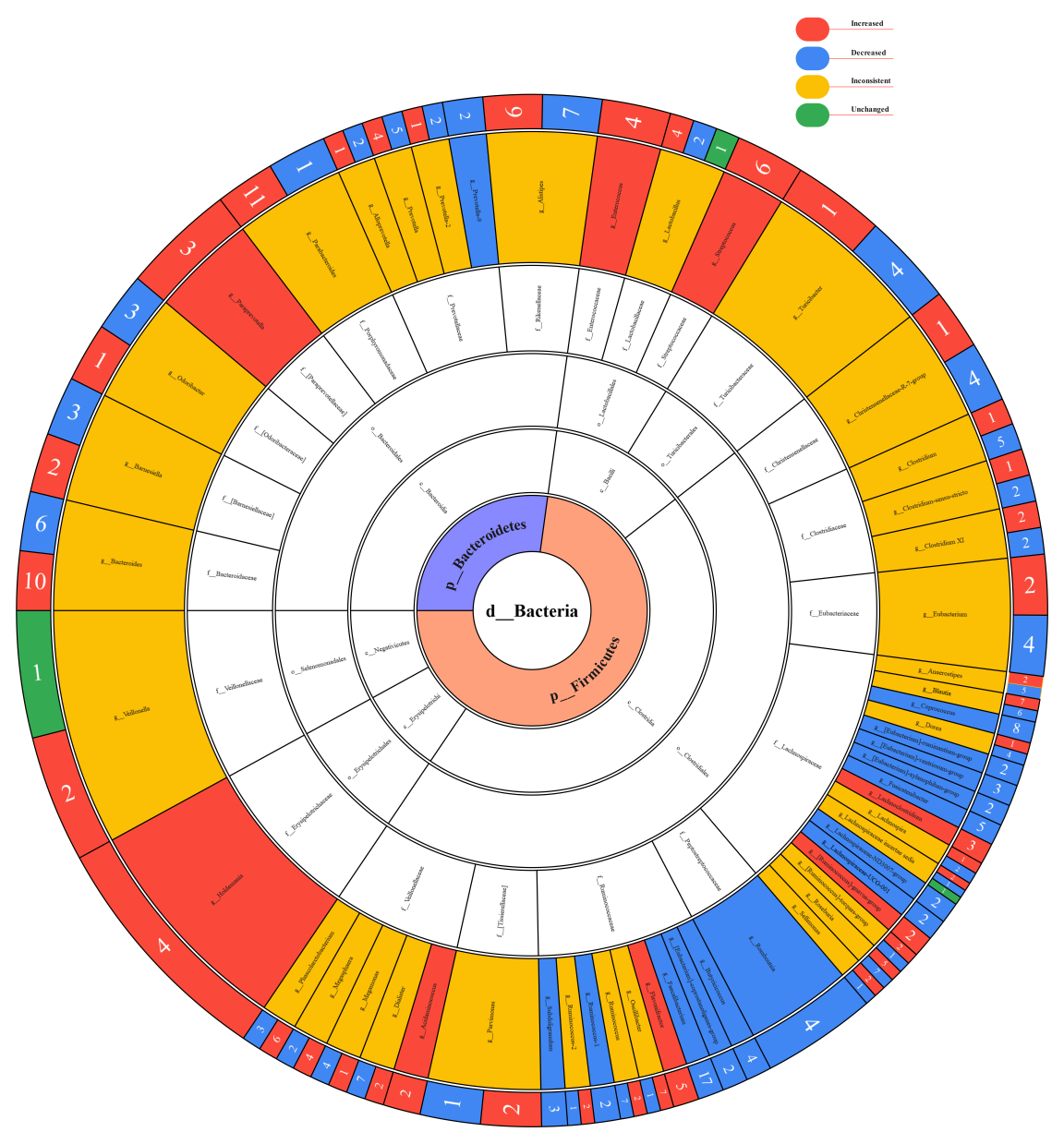
**

**
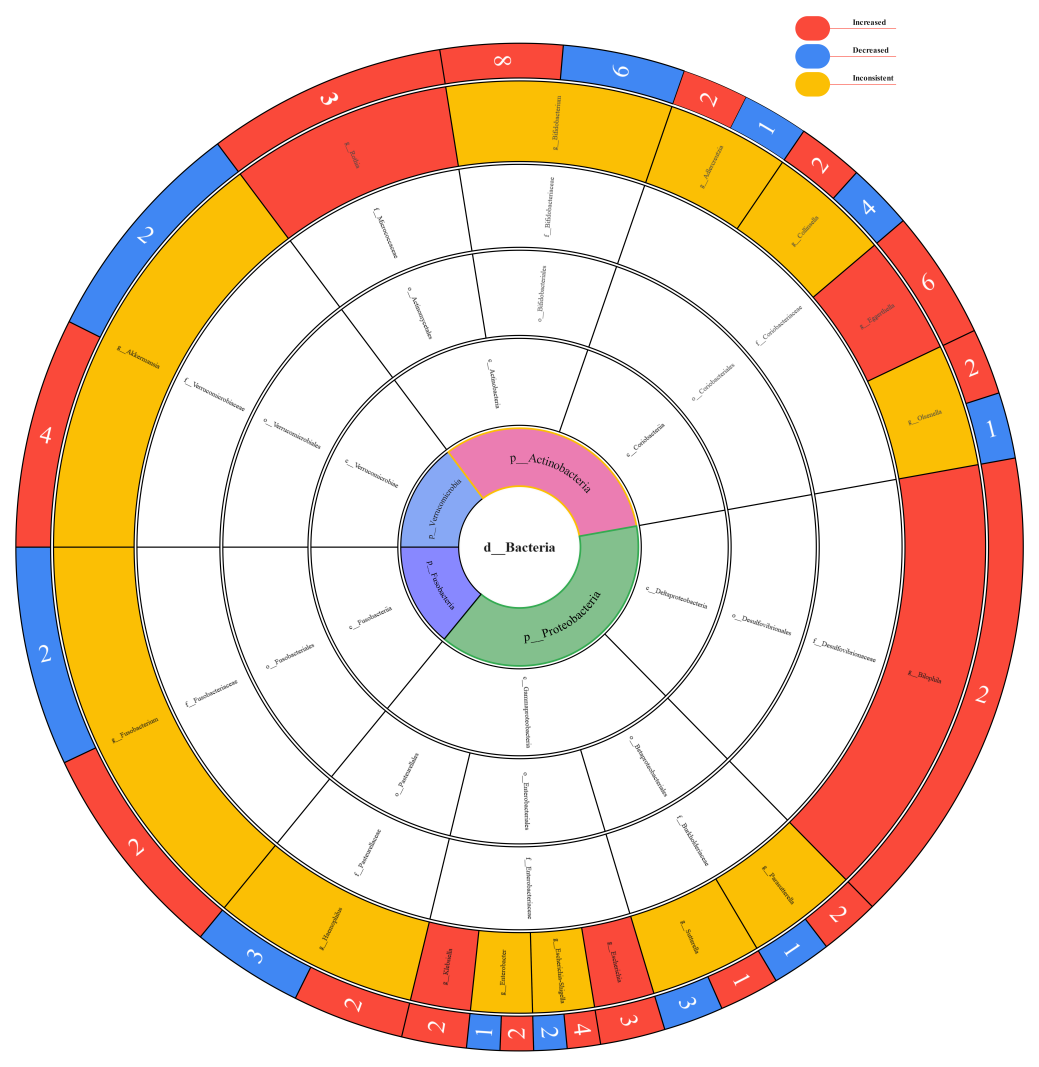
**

**Figure S2. Changes in Relative Abundance of Genus Level** **Reported by at Least 2 Studies**

Number in the outermost circle indicates amount of consistent studies

**
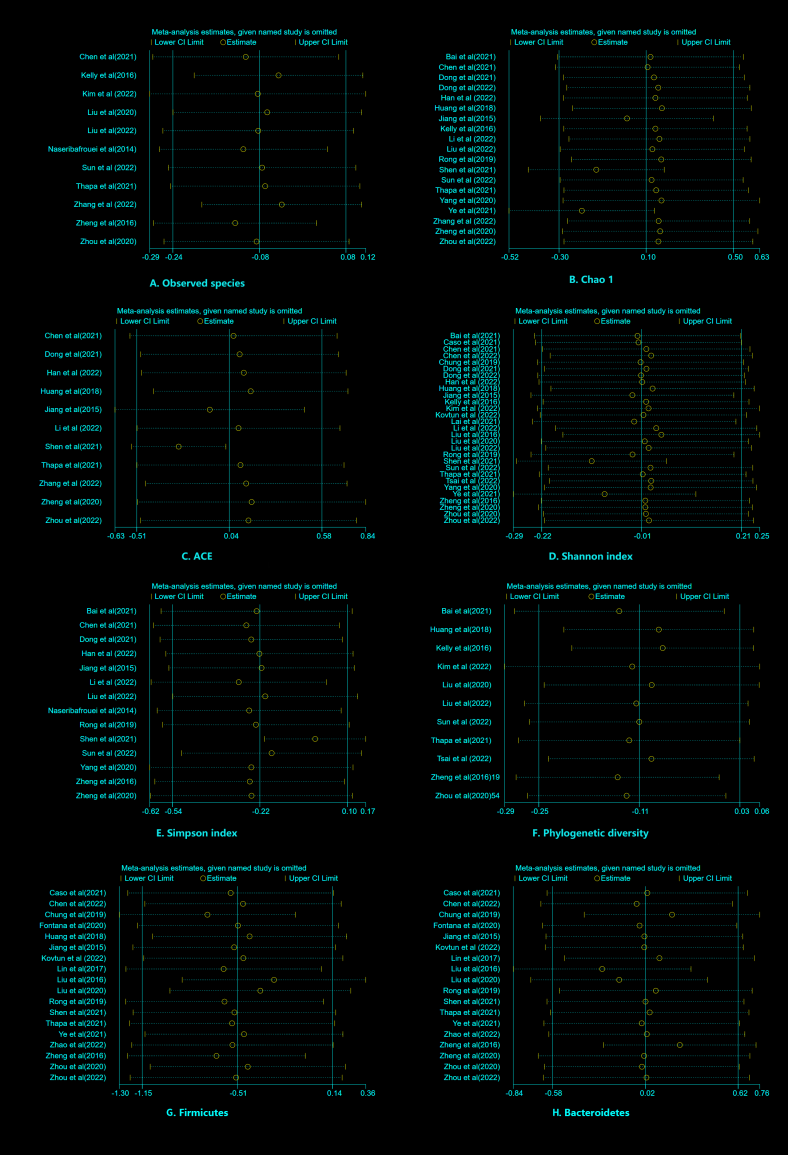
**

**Figure S3. Sensitivity analyses for the alpha diversity, the phylum Firmicutes and Bacteroidetes meta-analyses**

**
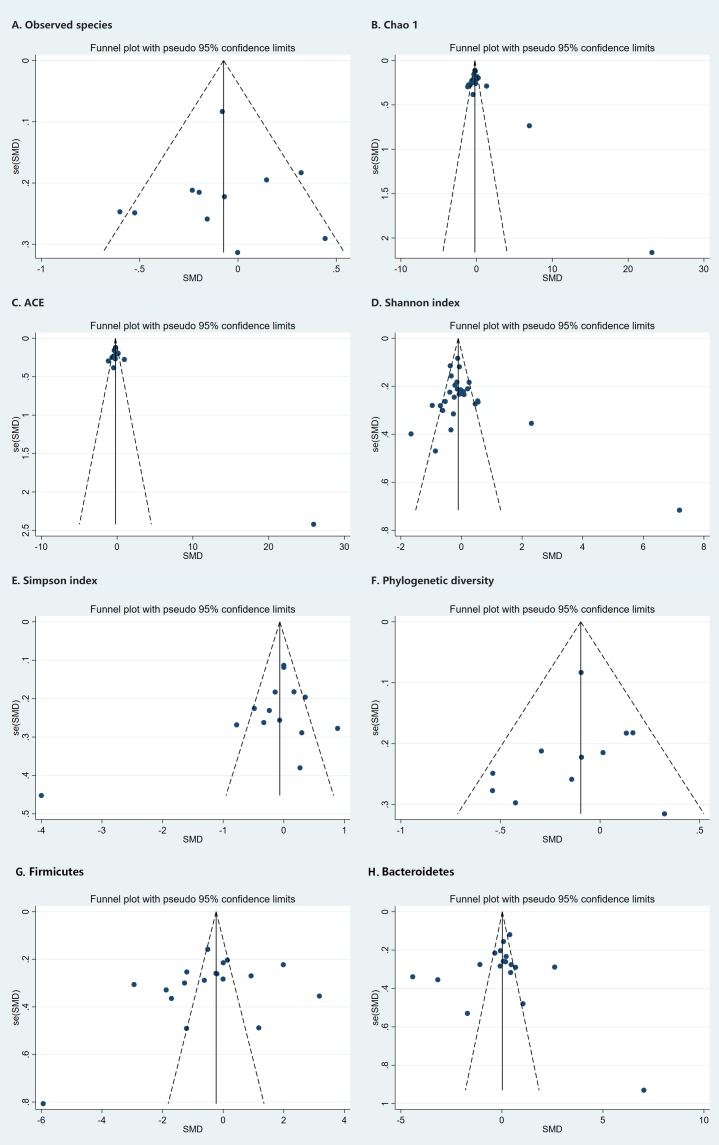
**

**Figure S4. Publication bias assessment for the alpha diversity, the phylum Firmicutes and Bacteroidetes meta-analyses**
